# Multifunctional Scaffolds and Synergistic Strategies in Tissue Engineering and Regenerative Medicine

**DOI:** 10.3390/pharmaceutics13060792

**Published:** 2021-05-26

**Authors:** Nicolas Muzzio, Sergio Moya, Gabriela Romero

**Affiliations:** 1 Department of Biomedical Engineering and Chemical Engineering, University of Texas at San Antonio, One UTSA Circle, San Antonio, TX 78249, USA; nicolas.muzzio@utsa.edu; 2 Center for Cooperative Research in Biomaterials (CIC biomaGUNE), Basque Research and Technology Alliance (BRTA), Paseo Miramon 182 C, 20014 Donostia-San Sebastian, Spain; smoya@cicbiomagune.es; 3 NanoBioMedical Centre, Adam Mickiewicz University, Wszechnicy Piastowskiej 3, 61-614 Poznan, Poland

**Keywords:** tissue engineering and regenerative medicine (TERM), biomaterials, scaffolds, multifunctional materials, combination therapy

## Abstract

The increasing demand for organ replacements in a growing world with an aging population as well as the loss of tissues and organs due to congenital defects, trauma and diseases has resulted in rapidly evolving new approaches for tissue engineering and regenerative medicine (TERM). The extracellular matrix (ECM) is a crucial component in tissues and organs that surrounds and acts as a physical environment for cells. Thus, ECM has become a model guide for the design and fabrication of scaffolds and biomaterials in TERM. However, the fabrication of a tissue/organ replacement or its regeneration is a very complex process and often requires the combination of several strategies such as the development of scaffolds with multiple functionalities and the simultaneous delivery of growth factors, biochemical signals, cells, genes, immunomodulatory agents, and external stimuli. Although the development of multifunctional scaffolds and biomaterials is one of the most studied approaches for TERM, all these strategies can be combined among them to develop novel synergistic approaches for tissue regeneration. In this review we discuss recent advances in which multifunctional scaffolds alone or combined with other strategies have been employed for TERM purposes.

## 1. Introduction

Tissue engineering and regenerative medicine (TERM) is a rapidly evolving field that applies the principles of engineering and life sciences dealing with the regeneration or replacement of damaged or diseased tissues and organs [1]. It is a multidisciplinary field that combines basic sciences such as cell biology, biomechanics, nanotechnology, polymer chemistry, materials science and bioinformatics, with applied medical sciences and engineering to promote tissue/organ repair or reconstruction [2]. The fabrication or regeneration of a tissue/organ replacement are very complex processes and often require the combination of several strategies, such as the development of multifunctional scaffolds, the delivery of growth factors (GFs) or other biochemical signals, cell and gene therapies, immunomodulation, and the use of external stimulus, i.e., electrical, or magnetic pulses. Tissue engineering and regenerative medicine have the potential to answer the increasing demand of organ replacements in a growing and aging world population [3], as well as to cope with the loss of tissues and organs due to congenital defects, trauma and diseases [4].

In tissues and organs, the extracellular matrix (ECM) is a crucial extracellular component that surrounds and acts as a physical environment for cells [5]. In addition to providing physical scaffolding for cell constituents, it contains intrinsic mechanical and biochemical cues that regulate cell phenotype and function and are required for tissue morphogenesis, differentiation, homeostasis, and response to injury [6,7]. The ECM is mainly composed of water, ions, organizational proteins like collagen, elastin and fibronectin, polysaccharides like hyaluronan and chondroitin sulfate, and proteoglycans [6,8]. However, each tissue has a unique ECM composition and topography, which interacts dynamically and reciprocally with the different cellular components, i.e., epithelial, endothelial, adipocyte, fibroblast cells have distinct ECMs [9]. For instance, the elastic properties of structural components of the ECM and the continuous bidirectional cell-matrix interaction, mainly through collagen-binding β1-integrins, regulate the hydrostatic forces of tissues [10,11]. As a fundamental mediator for most of intracellular events, abnormalities in the ECM, such as anomalous amount or activity level of a matrix component, act as driving force in the development of mammalian diseases [8]. Given the importance of the ECM, it is used as model guide for the design and elaboration of scaffolds and biomaterials, which often try to emulate the ECM natural properties and stimuli responsiveness [7]. These properties and cues can be physicochemical, i.e., stiffness, topography, charge density, wettability; or biochemical, i.e., presence of growth factors, hormones, ions. Such properties determine the interaction of the biomaterial with the biological environment [12]. The engineering of new materials with controlled properties mimicking cellular and tissue characteristics is of particular interest for the biomedical field for the development of implants and medical devices, but also as a platform for fundamental studies at the cell and cell colony level [13].

**Scheme 1:**
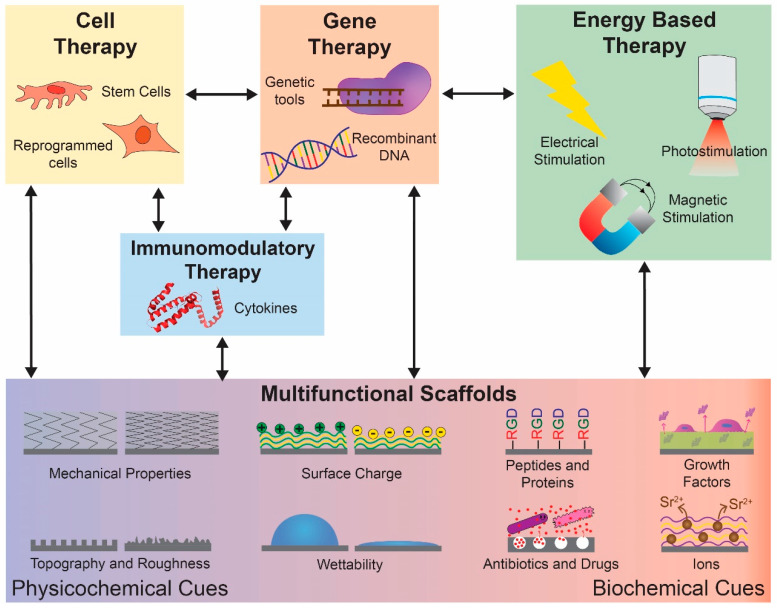
Overview of different strategies and their combination with multifunctional scaffolds for tissue engineering and regenerative medicine.

Although the development of multifunctional scaffolds and biomaterials is one of the most frequent approaches for TERM, several other strategies can be applied alone or in combination to achieve the goals of TERM. Autologous (cell source is the patient) and allogenic cells (other cell source) can be used as a therapy for functional regeneration of cells and tissues [14]. Gene therapy is often utilized to introduce genes into living cells for editing, replacing, or repairing the expression of specific proteins in damaged tissues or organs [15], or simply to condition cells for further use in cellular therapy. External stimulation strategies such as electrical [16], magnetic [17] and photo/optical [18,19] can be also applied to modulate cell behavior, induce tissue regeneration and remotely control drug delivery or therapeutic actions. All these strategies can be combined among them and with biomaterials and multifunctional scaffolds to develop novel synergistic approaches for tissue regeneration (Scheme 1).

Several reviews have already addressed independently the different stimulus, biomaterials properties and strategies used in tissue engineering [20,21]. However, in the last years, special attention has been allocated to the combination of different strategies, physical and biological or more than one biological/physical stimuli; thus, there is an imminent need for a review collecting synergistic strategies and techniques being studied for TERM. In this review we will summarize recent strategies in which multifunctional scaffolds alone or combined with other strategies have been employed for tissue engineering purposes. In Table A1, we have compiled the abbreviations used in this review for the reader’s convenience.

## 2. Multifunctional Scaffolds

Biomaterials with only one variable cue/property that mimic single aspects of the ECM are essential for understanding the effect of that property on isolated cells or on cell colonies behavior [22]. However, combining different properties in engineered multifunctional materials and scaffolds is necessary to better mimic the complex ECM and understand the interplay between different cues and their influence on cell behavior. In this section, we briefly discuss the different physicochemical and biochemical properties in a scaffold and their influence on cell functions.

### 2.1. Physicochemical Cues

#### 2.1.1. Mechanical Properties

Cells are able to sense external mechanical signals from other cells and from the ECM, and transduce these stimuli into biochemical and electrical signals regulating different cellular processes such as adhesion, migration, proliferation and differentiation [23,24]. From brain to bone, each tissue and organ present its own mechanical properties [25]. Cell adhesion, morphology and proliferation are highly dependent on the mechanical properties of the substrate on which cells are growing [26]. Moreover, cells on thin, soft coatings, i.e., polymeric films deposited on a hard material, can ‘feel’ an underlying stiffer substrate and sense a complex stiffness, i.e., the combination of the stiffness of both upper soft material and the hard substrate underneath [27,28]. Though most cell types present better adhesion properties on stiff substrates (above 100 kPa), some cell types such as neurons can adhere on soft ones (below 3 kPa) [29]. Certain cell types are able to follow gradients in the stiffness of ECM in a process called durotaxis, which can be used to guide cell migration for tissue engineering purposes [30]. Moreover, it has been demonstrated that stem cell differentiation is largely affected by the substrate’s stiffness, a neural lineage on soft substrates and an osteogenic one on stiffer substrates being more likely [31]. As cell types may respond differently to the same mechanical stimuli, an engineered biomaterial intended to replace a tissue should resemble the mechanical properties of native tissues.

#### 2.1.2. Roughness and Topography

The organization of the ECM in the different tissues of human body is frequently hierarchical with different spatial resolution: nanoscale (features below 100 nm), micro/submicroscale (features from 100 to 1 µm) and macroscale (features larger than 1 µm) [32]. Each tissue presents specific structures associated with its specific function in the body. For instance, unidirectional alignment of myotubes is required for maximal generation of a contractile force in skeletal muscle [33]. Different topographic surface properties such as roughness, topological cues (e.g., pores, gratings, grooves/channels, pillars, or pits) and curvature can affect cell behavior in a cell-type specific manner [34,35]. The size and spatial arrangement of this features is also an important factor. For example, substrates with nano to micro roughness gradients revealed that endothelial cells spread and proliferate in highly rough regions where smooth muscle cells present a shrunken morphology and inhibited growth [36]. Hybrid micro/nanostructures with nanorods and micropatterns synergistically enhanced cell adhesion, proliferation and osteogenic differentiation of human bone marrow stromal cells in comparison with the surface structures of nanorods or micropatterning alone [37]. Cell movement or/and collective cell colony migration can be controlled by surface topographical cues and scaffolds displaying nanofibers [38], microridges [39] and porous channels [40]. Even local asymmetric topographical ratchets can induce cell polarization and direct cell motion (ratchetaxis) [41]. Cells can sense substrate stiffness and topography and trigger different adhesion, proliferation, or differentiation responses. However, “cells cannot feel what they cannot hold on to” [42], and a proper binding between ECM adhesive proteins (e.g., fibronectin, laminin, vitronectin, etc.) and cells’ integrins and other receptors is necessary for cell interaction with the ECM and adhesion to the substrate [43]. Cell-surface nanotopography interactions are dependent on the nanoscale protein interface [44]. Surfaces with topographical features of similar size to the physical dimensions of proteins may determine the type and amount of proteins adsorbed and their conformation [34]. For instance, fibrinogen’s adsorption kinetics and orientation can be controlled on semicrystalline nanostructures [45].

#### 2.1.3. Wettability, Polarity and Surface Energy

Though water contact angle is not a good general predictor of biological response (e.g., cell adhesion) to materials [46], its simple and accessible determination makes it a commonly reported parameter to correlate cell behavior with materials characteristics. It has been suggested that surfaces with moderate wettability, i.e., a contact angle around 85°, are optimal for promoting cell adhesion because they promote a proper state of the adhesion-mediating ECM proteins [47]. The quantity of adsorbed proteins can be relatively large on highly hydrophobic surfaces; however, high protein-surface and intraprotein interactions may produce denaturation or impede cells’ remodeling. On the other hand, on highly hydrophilic surfaces, weak protein–substrate forces may render a labile adsorption of adhesion proteins, limiting cell attachment. Experiments of adsorption and exchangeability of fibronectin with bovine serum albumin or serum on polyelectrolyte multilayers with different surface properties (hydrophobicity/hydrophilicity and surface charge) using radiolabeled proteins revealed that cell adhesion is strongly dependent on surface–protein interactions [42,48]. Along with contact angle, another related physicochemical property of the material’s surface which is often reported and related to cell behavior is surface energy, i.e., the excess energy at the surface of the material compared to the bulk [47,49]. Comparing surfaces of similar roughness, more fibronectin adsorption has been reported on higher surface energy substrates [50]. Different studies have shown a positive direct correlation between cell adhesion and surface energy [51,52]. Other authors have reported a positive dependence of cell adhesion on the polar component of the surface energy [53,54].

#### 2.1.4. Surface Charge

The surface charge of the material is another important factor determining material-protein interactions and subsequently, material–cell interactions. Protein adsorption depends on hydrophobic interactions, but also, on electrostatic interactions [55]. Some research works indicate that positively charged surfaces support cell adhesion better than negatively ones [12]. For example, surfaces with varying zeta-potentials were obtained using self-assembled monolayers (SAMs) and mouse embryonic fibroblast adhesion was better on the most positive ones [56]. This could be related to the net negative charge of cell membrane (affecting mainly the first moments/phase of material–cell interaction) and of most of the cell adhesion mediating ECM species that would preferentially absorb on positively charged interfaces [47]. However, it has been demonstrated that, despite having a net negative charge, fibronectin positively charged regions can be attracted and deposited on negative carboxylate terminated SAMs [57]. The spatial orientation and conformation of the adsorbed cell adhesion-promoting proteins is more important than their amount. Protein adsorption is a complex process in which the interplay of different factors, such as surface properties (e.g., topography, surface energy and surface charge), protein physical properties (e.g., flexibility and concentration) [58,59] and even cell activity (e.g., protein remodeling/reorganization and fibrillogenesis) [60,61], determine cell fate.

### 2.2. Biochemical Cues

#### 2.2.1. Growth Factors (GFs)

GFs are biologically active molecules secreted by the organisms that control cellular responses such as mitosis, mobility or differentiation [62]. They act on targeted cells through specifically binding transmembrane receptors that convey their signals to intracellular components. GFs play essential roles in embryonic development [63], wound healing [64] and cancer progression [65]. As GFs play key roles in tissue development and regeneration, GF-based therapy for TERM generated initially much enthusiasm [66]. However, limited success in clinical trials have been obtained. The poor interactions of some GFs with the ECM and the short-term burst-type effect due to diffusion and proteolysis limit their action [67]. Therefore, supraphysiological and repeated doses are required for therapeutic benefit, which lead to side effects such as ectopic tissue formation and increased cancer risk. Sophisticated material carriers able to control the spatiotemporal bioactivity of GFs are attractive for increasing their therapeutic effect. Micro- and nanoparticles, injectable hydrogels, scaffolds, among other materials have been applied as delivery systems to provide improved stability and controlled release of GFs [68].

Different strategies are being developed to mimic the natural dynamic microenvironments of tissue formation and repair by the local delivery of different exogenous GFs in appropriate ratios, physiological/pharmacological concentrations and following specific spatiotemporal patterns [69]. For example, bone formation is a complex process, and the concerted function of angiogenic factors in the early phases promotes vascularization, and osteogenic factors during the whole bone generation process are required [70]. In reference [71], the authors encapsulated bone morphogenetic proteins (BMP) in poly(lactic-co-glycolic acid) (PLGA) microspheres which are embedded in a poly(propylene) scaffold surrounded by a gelatin hydrogel loaded with vascular endothelial growth factor (VEGF) for the sequential delivery of GFs that promoted vascularization and bone formation. In another example, Liu et al. encapsulated nerve growth factor and glial cell line derived neurotrophic factor in poly(d, l-lactic acid) (PLA) and PLGA nanofibers, respectively, for a sustainable and tunable dual release of GFs from scaffolds with potential applications in peripheral nerve regeneration [72]. GFs can also be used as chemoattractant agents to accelerate and guide cell migration to the scaffold or defective tissue [73].

#### 2.2.2. Antibiotics and Drugs

With the widespread occurrence of nosocomial infections and the emergence of new bacterial strains, the inhibition of bacterial adhesion/colonization is of utmost importance in the design of biomaterials for tissue engineering [74]. The encapsulation of antibiotics within the scaffold and their localized release is one of the most common strategies to prevent infections. Several approaches for antibiotic loading/encapsulation in surfaces and scaffolds have been developed, involving porous materials [75], layer-by-layer assembly of polyelectrolytes with antibiotics [76] or antibiotic-containing supramolecular complexes [77], hydrogels [78] or polymer brushes [79].

#### 2.2.3. Ions

Some elements, even as traces, are essential components of the human body and provide many advantages for its biological functions [80]. For example, Zinc (Zn) and Calcium (Ca) play a role in protein structure, Ca and Silicon (Si) in bone structure, Manganese (Mn) and Magnesium (Mg) in nucleic acid structure and Iron (Fe) and Copper (Cu) are fundamental in electron transfer processes [81]. Other elements, though not essential, can improve organism functionality. These ‘beneficial’ elements often share chemical similarities and characteristics with essential elements, and affinity for the same chemical sites [80]. Using these ions in tissue engineering and biomaterials could have a positive impact on cells and improve regeneration. For example, Strontium (Sr^2+^) resembles Ca^2+^ and can be found bonded to phosphates in bone with its ranelate salts having a protecting effect against osteoporosis [82]. It has been proposed that the release of Sr^2+^ from Titanium (Ti) implants impacts positively on osseointegration, as it promotes osteogenic differentiation of mesenchymal stem cells (MSCs) [83] and increase proliferation and expression of osteogenic markers in preosteoblastic cells [84]. Several ways can be followed for the delivery of ions from a scaffold, for example using metal ions crosslinked hydrogels [81], polymer brushes [85], nanoparticles embedded in matrix/scaffold [86], mesoporous materials [84] and mineral coatings such as Sr-substituted hydroxyapatite (HA) [87].

#### 2.2.4. Peptides and Proteins

ECM peptides and proteins are diverse both in structure and function. Structural proteins such as collagen (Col) or elastin provide strength, some proteoglycans and hyaluronic acid sequester water and divalent cations for space-filling functions, whereas other proteoglycans bind GFs for reservoir and release, phosphatidylcholine lipids and mucinous glycoproteins contribute to biolubrication, while other glycoproteins such as fibronectin and laminins provide signals for cell adhesion or differentiation, and proteases promote ECM remodeling [88,89,90,91,92]. Fibronectin, Col or peptides that promote cell adhesion such as the amino acid sequence arginine-glycine-aspartate (RGD) are often covalently bounded to hydrogels and scaffolds in several tissue engineering strategies [93]. For example, RGD-immobilized macro porous alginate scaffolds were more effective in promoting cell adherence, preventing cell apoptosis and accelerating cardiac tissue regeneration in comparison with unmodified scaffolds [94]. Antimicrobial peptides, i.e., short peptides with a broad range of antibacterial activities, can be incorporated into biomaterials to prevent infections [74]. Several of these peptides can modulate the immune response of the host and promote pathogen clearance [95]. Scaffolds can be also functionalized with enzymes having antimicrobial properties, such as lysozyme that cleaves peptidoglycan component of bacterial cell walls, and biofilm-dispersing/matrix disruptive enzymes, such as DNase I and dispersin B that produce biofilm detachment [96]. As most of the time scaffolds and constructs are not intended as permanent implants, gaining control of their biodegradability is a key factor to successful tissue regeneration [97]. In that vein, Kim et al. developed chitosan-lysozyme scaffolds for bone tissue engineering and they were capable of controlling the degradation rate of the hydrogel by varying the enzyme concentration [98].

## 3. Other Strategies in Tissue Engineering and Regenerative Medicine

Apart from functionalized scaffolds and biomaterials, several approaches can be applied in TERM to target different aspects of cell and tissue growth and regeneration. In this section some of these strategies will be discussed.

### 3.1. Cell Therapy

Cell therapy may be defined as the prevention or treatment of a disease or injury by the administration of cells that have been selected, multiplied, differentiated, genetically modified and/or pharmacologically treated ex vivo (i.e., outside the body) [99]. Autologous and allogeneic stem, gene engineered, differentiated or adult cells may be used, each approach having its own advantages and disadvantages [14]. For example, autologous cultured chondrocytes on a porcine Col membrane and allogeneic cultured keratinocytes and fibroblast in bovine Col are approved treatments by the U. S. Food and Drug Administration for knee’s cartilage defects and wounds of the oral soft tissue, respectively [14]. Due to their self-renew capacity and their ability to differentiate into various specialized cell types under certain environmental conditions, most of the research in cell therapy centers around stem cells [100]. MSCs play a critical role in growth, wound healing and replacement of cells that are lost through physiological and pathological conditions, and consequently, are effective in the treatment of tissue injury and degenerative diseases [100]. One of the major limitations in cell therapy is the actual delivery of the cells to a targeted site, and to address this scaffold-free and scaffold-based approaches are being studied and developed [101]. In scaffold-free delivery approaches, mainly three concepts are applied: single cells, cell sheet engineering or microtissue technology [102]. Single cells can be directly administered via injection in the affected tissue or systemically via intravenously or intracoronary injection. For example, systemically injected MSCs are found to migrate specifically to damaged and inflamed tissues. However, many of the intravenously administered cells get trapped in the lungs, liver, or spleen. As an alternative to single-cell delivery, confluent monolayers of cells grown in culture dishes (cell-sheets) or 100–500 µm in diameter cell reaggregates generated from dispersed cells (microtissues) can be placed onto the target tissue. On the other hand, in scaffold-based approaches, engineered biomaterials such as porous scaffolds, nano-microparticles or injectable hydrogels act as cell carriers, providing biological signals, protection, homing and retention at the targeted tissue [101]. Multifunctional scaffolds for cell therapy may be used in combination with drug/GFs release, gene delivery strategies and electromagnetic stimulation [103,104].

### 3.2. Gene Therapy

Gene therapy may be defined as the addition, correction, or removal of specific genetic sequences in targeted cells, or cells that will be delivered to a patient with the ultimate purpose of preventing or treating a particular disease [99]. Different genome-editing tools such as zinc finger nucleases, clustered regularly interspaced short palindromic repeats -associated protein 9 (CRISPR/Cas9) system, PiggyBac transposon, transcription activator-like effector nucleases (TALENs) etc., can be employed to precisely manipulate human genome to achieve a therapeutic effect [105]. A critical component of gene therapy is the carrier, vehicle, or vector to healthy deliver the gene/genome-editing tools to the cells. Though vectors are usually viral, many new techniques and materials are being developed to avoid the use of viral material which induce innate and adaptive immune responses that obstacle their use in patients [106]. Rapid and spatially localized gene delivery has been achieved by association of plasmid DNA with peptides and iron oxide magnetic nanoparticles (MNPs) by application of magnetic fields [107]. Gene-editing tools such as CRISPR/Cas9 plasmids and PiggyBac can be delivered using lipid-polymer hybrid nanoparticles, PLGA nanoparticles and covalently stabilized poly (β-amino ester) nanoparticles [108,109,110]. Gene-activated scaffolds are being developed for scaffold-mediated delivery of DNA and RNA in combination with GFs release and MSCs for bone and cartilage tissue engineering [103,111]. Gene therapy can be used to assist bone repair by the enhancement of VEGF expression for vascularization improvement, by inducing BMP or transforming growth factor β1 (TGF-β1) expression for enhancement of osteogenic commitment and mineralization, or by targeting inhibitory genes of osteogenesis. For instance, bone-mimicking collagen hydroxyapatite scaffold loaded with chitosan nanoparticles carrying plasmid DNA genes encoding osteogenic BMP-2 and angiogenic VEGF proteins synergistically increased new vessel formation and bone repair in a critical-size calvarial defect model [112].

Scaffold-based gene therapy can be also employed for neural tissue engineering applications. Nguyen et al. used a biodegradable, three-dimensional, aligned scaffold composed of poly (ε-caprolactone-*co*-ethyl ethylene phosphate) electrospun nanofibers and collagen hydrogel for delivery and nonviral transfection with miR-222, a microRNA that participates in controlling local protein synthesis at distal axons [113]. The implantation of microRNA containing scaffolds significantly enhanced axon regeneration and remyelination in an in vivo rat model of spinal cord injury, in comparison with animals implanted with plain scaffolds. Gene activated biomaterial consisting of a collagen-chondroitin sulfate scaffold loaded with polyethyleneimine carrying a plasmid encoding for stromal derived factor-1 alpha (a chemokine that induces the expression of VEGF and guides endothelial vascularization) can enhance proangiogenic response, which is needed during wound healing process [114].

### 3.3. Immunomodulatory Therapies

The immune system plays an essential role in directing wound healing and tissue repair and regeneration [115]. For instance, immune response to a myocardial infarct is essential to preserve tissue integrity and avoid a fatal cardiac rupture, but may lead to the formation of a rigid scar tissue, causing structural changes and compromising tissue functionality [116]. Therefore, modulating the body’s own endogenous processes is necessary to prevent tissue/organ damage and to enhance regeneration or recovery [117]. Manipulating immunologic response may improve acute and chronic organ dysfunction. Moreover, immune state is highly variable and will impact the outcome of tissue-engineered products in the clinic [115]. Cells, cytokines, and biomaterials can be utilized for immune response modulation in TERM. Mesenchymal stromal cells are able to adjust the adaptive and innate immune response providing a suitable milieu for tissue recovery and can also differentiate into various types providing cell replacement [118]. Different cytokines can assist tissue repair acting as homing factors for cell recruitment, enhance proliferation, guide differentiation and are also able to regulate immune responses such as inflammation [119,120]. Along with the intrinsic properties of biomaterials (shape, surface charge, etc.) that are able to influence immune responses (by, for example, polarizing macrophages and dendritic cells toward either an inflammatory or wound healing phenotype), cells and cytokines can be combined with scaffolds to achieve immunomodulation capacity for TERM [121].

### 3.4. Electrical, Magnetic and Optical Stimulation

Endogenously generated bioelectric currents play a fundamental role in different biological processes such as embryogenesis, wound healing, tissue repair and remodeling, as well as in the physiological function of nerve, muscle and glandular tissues [122]. The biophysical mechanisms by which cells sense and transduce electrical cues into biochemical and biological signals is unclear, but it may involve modulating the conformation and functionality of cell membrane proteins like enzymes, membrane–receptor complexes and ion-transporting channels, leading to altered intracellular concentrations of certain ions, such as Ca. Electrical stimulation is a promising tool/therapy in tissue engineering as can affect cell adhesion, alignment, proliferation, differentiation, migration (galvanotaxis) and apoptosis [123]. For instance, electrical stimulation has been shown to accelerate nerve regeneration and motor and sensorial functional recovery in rats [124], and promote bone healing after fracture in both animal and clinical studies [125]. Different methods can be used to apply an electrical stimulation [126]. In direct coupling, the electrode is in contact with the cell culture or implanted into the patient, which can lead to tissue damages. Indirect coupling refers to noninvasive methods in which a homogenous magnetic field is created between two parallel layers of metal or carbon (capacitive coupling), or controlled magnetic fields are generated by coils (inductive coupling). In this vein, electrically conductive multifunctional scaffolds can act as new means to deliver well-controlled electrical stimulation [126]. For example, polypyrrole/silk fibroin conductive composite scaffolds fabricated by 3D bioprinting and electrospinning effectively promote axonal regeneration and remyelination in vivo [127].

Pulsed electromagnetic fields have also been found to accelerate wound healing by modulating cell processes such as proliferation, apoptosis, differentiation, migration and DNA replication and expression [16]. The proposed mechanisms of the regenerative effects of magnetic stimulation are associated with ionic transport, and protein and growth factor metabolism [124]. As sometimes traditional methods are not sufficient to repair certain tissue injuries or defects, biomaterials can be used to improve therapeutic effects. Magnetic tissue engineering aims to develop complex systems in which magnetic materials, such as MNPs, are used as remotely controlled mutidimensional tools [17]. For instance, using alternating magnetic fields and histeresis power loss from MNPs, remote neural excitation can be achieved through the activation of the neuron’s heat-sensitive receptors [128] or the local delivery of drugs [129]. In another approach, MNPs and mechanical stimulation induced by magnetic fields were shown to promote osteogenic and chondrogenic differentiation of human stem cells in the corresponding supplemented media [130]. Magnetic materials can be incorporated into different scaffolds such as hydrogels in order to provide them with physical anysotropy and use them as remote magnetomechanic actuators to guide cell behavior [131].

Apart from electrical and magnetic stimulation, phototherapy/optical stimulation are promising strategies in tissue engineering. For instance, optical stimulation of neuronal tissue is significantly more focused than electrical stimulation, which would enable the activation of more discrete, independent populations [18]. Neurons can be directly stimulated with infrared light without modification or with visible light in neurons genetically modified to express light-sensitive molecules to enable responsiveness (optogenetics). Laser phototherapy has presented promising results in tissue engineering by influencing the proliferation and differentiation of human stem cells from exfoliated deciduous teeth [19]. Phototherapy can also be used in combination with scaffolds in photodynamic and photothermal applications in order to treat/prevent bacterial infection an fight cancerous tumors [132].

## 4. Multifunctional Scaffolds in Tissue Engineering

As discussed above, to mimic the complexity of native ECM for TERM, it is necessary to fabricate multifunctional scaffolds or biomaterials capable not only to emulate ECM natural properties, but also to respond to specific stimulus from the cellular microenvironment. In this section, we summarize the most relevant contributions encompassing the synergistic effects of different physicochemical and biochemical cues in multifunctional scaffolds for TERM combination therapy.

### 4.1. Synergistic Approaches among Physicochemical Cues

**Figure 1:**
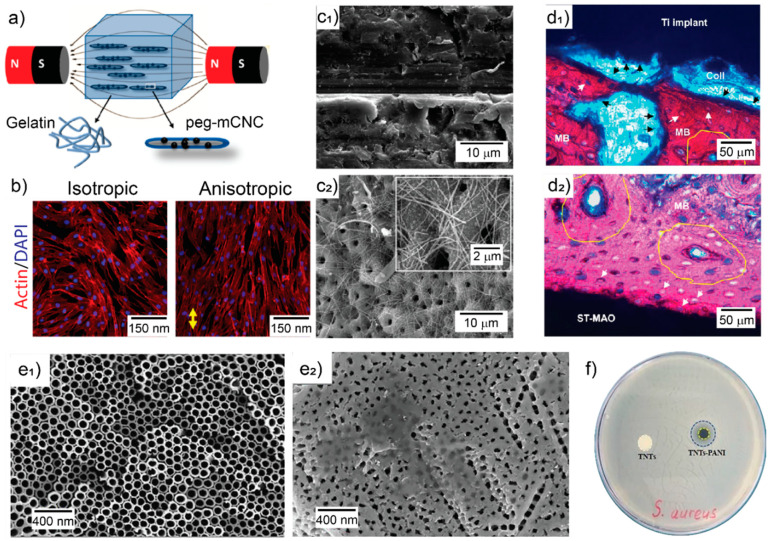
Scaffolds combining synergistic physicochemical cues for TERM. (**a**) Schematic representation of anisotropic hydrogel preparation and cross-linking under the influence of a uniform magnetic field. Rod-shaped nanocrystals coated with MNPs, polydopamine and polyethylene glycol were employed to generate a gelatin hydrogel with directional microstructure and anisotropic mechanical properties. (**b**) Effect of isotropic and anisotropic hydrogels on human adipose tissue derived stem cells alignment after 3 days of culture (red, cytoskeleton; blue, nucleus). (**c_1_**,**c_2_**) SEM images of Ti implant and steam hydrothermal treated microarc oxidation coated Ti (ST-MAO), respectively. (**d_1_**,**d_2_**) Bone histology and histomorphometry around the Ti and ST-MAO implants, respectively, after 12 weeks of healing. (MB) mineralized bone; (black arrow) osteoblasts; (white arrow) osteocytes; (Coll) collagen birefringence; (yellow ring) osteon. (**e_1_**,**e_2_**) Field Emission-SEM TNTs and TNTs coated with PANI, respectively. (**f**) Antibacterial effect showing zone of inhibition on *S. aureus*. (**a**,**b**) adapted with permission from [133], American Chemical Society, 2019. (**c_1_**–**d_2_**) adapted with permission from [134], American Chemical Society, 2015. (**e_1_**–**f**) adapted with permission from [135], Elsevier, 2018.

Through the combination of appropriate mechanical properties and topographical cues, S. Araújo-Custódio et al. developed an injectable hydrogel composed of gelatin and rod-shaped magnetic cellulose nanocrystals coated with polydopamine and polyethylene glycol (Figure 1a). By varying the nanoparticle concentration, they were able to tune gel stiffness, and by applying a low static magnetic field during crosslinking they generated a biomaterial with directional microstructure and anisotropic mechanical properties. This hydrogel induced the directional growth of seeded human adipose tissue derived stem cells (Figure 1b) [133].

Developing facile ways of tuning interfacial properties of materials is important for modulating cell behavior. In reference [134], Zhou et al. reported the synergistic effects of hierarchical topography and surface chemistry of modified Ti implants for bone tissue engineering. The authors generated a porous coating of Ca, Phosphorus (P), Si and Sodium (Na) on Ti using microarc oxidation (MAO). Bioactivity was significantly improved introducing hydroxyl functional groups and increasing the apatite-inducing ability of the MAO coating by steam-hydrothermal treatment. The resulting surface exhibited a hierarchical structural complexity with microscale pores (2–5 µm) and nanoscale HA wires and anatase dots (Figure 1(c_1_,c_2_)). This hierarchical topography along with the improved surface chemistry resulted in a synergistic effect that enhanced bone regeneration and bone–implant contact (Figure 1(d_1_,d_2_)), and increased biomechanical push-out force of the modified Ti implants in rabbit tibia in comparison with the bare Ti implants. Likewise, in reference [136], Metwally et al. tailored the surface chemistry and potential of polycaprolactone (PCL) fibers by simply changing the voltage polarity from positive to negative during electrospinning. The fibers produced with negative voltage polarity doubled the surface potential of fibers produced with positive voltage, correlated with a decrease in oxygen content at the fiber surface. Human osteoblast-like cell line MG-63 presented increased proliferation, Col-like fibers formation and filopodia formation on PCL fibers with higher surface potential. In order to emulate the mechanical and functional properties of natural bone [137], Kaur et al. used the freeze drying technique to fabricate polyvinyl alcohol (PVA)-carbon nanotubes (CNTs) nanocomposite scaffolds. Carboxylic acid functionalized CNTs were used to reduce the tendency of agglomeration of CNTs, and their concentration was varied to tune physicochemical cues. CNTs reinforcement enhanced the mechanical properties of the nanocomposite scaffolds. It also increased the adsorption of proteins via electrostatic interactions between proteins and the hydrophilic functional groups of CNTs. In comparison to control well plates and pure PVA scaffolds, the reinforced materials enhanced MG-63 osteoblast adhesion, promoted cell proliferation and differentiation, and reflected an increased alkaline phosphatase activity, matrix mineralization and Col secretion.

Electroconductive biomaterials can provide an appropriate cell microenvironment and cell guidance, thus being beneficial in cardiac, muscle, nerve or bone tissue engineering [138]. In reference [135], the authors developed titanium oxide nanotubes (TNTs) via electrochemical anodization process followed by polyaniline (PANI) functionalization using cyclic voltammetry at low temperature (Figure 1(e_1_,e_2_)). The TNTs showed large surface area to volume ratio and increased electrical conductivity, hydrophilicity and in vitro mineralization after coating with the electroactive conductive polymer PANI. The bioinspired TNTs/PANI composite showed effective antibacterial properties against *E. coli* and *S. aureus* (Figure 1f). MC3T3-E1 preosteoblast cells seeded on TNTs/PANI scaffolds presented enhanced attachment, proliferation and expression of osteogenic-related markers (alkaline phosphatase activity and collagen type 1 secretion) in comparison to bare Ti and TNTs. Moreover, in reference [139], Magaz et al. developed an electroconductive scaffold with the fibrillary topography of the native ECM for nerve tissue engineering. Electrospun silk fibroin scaffolds were functionalized with poly (3,4-ethylenedioxythiophene)-polystyrene sulfonate (PEDOT−PSS) and their conductance was further boosted by dimethyl sulfoxide etching. Both, conductivity and protein absorption capacity, increased with the concentration of PEDOT-PSS. The electroconductive scaffolds were biocompatible with analogue NG108-15 neuronal cells and presented better biological outcome in terms of cell proliferation and neuronal differentiation than on unmodified silk.

### 4.2. Synergistic Approaches among Biochemical Cues

**Figure 2:**
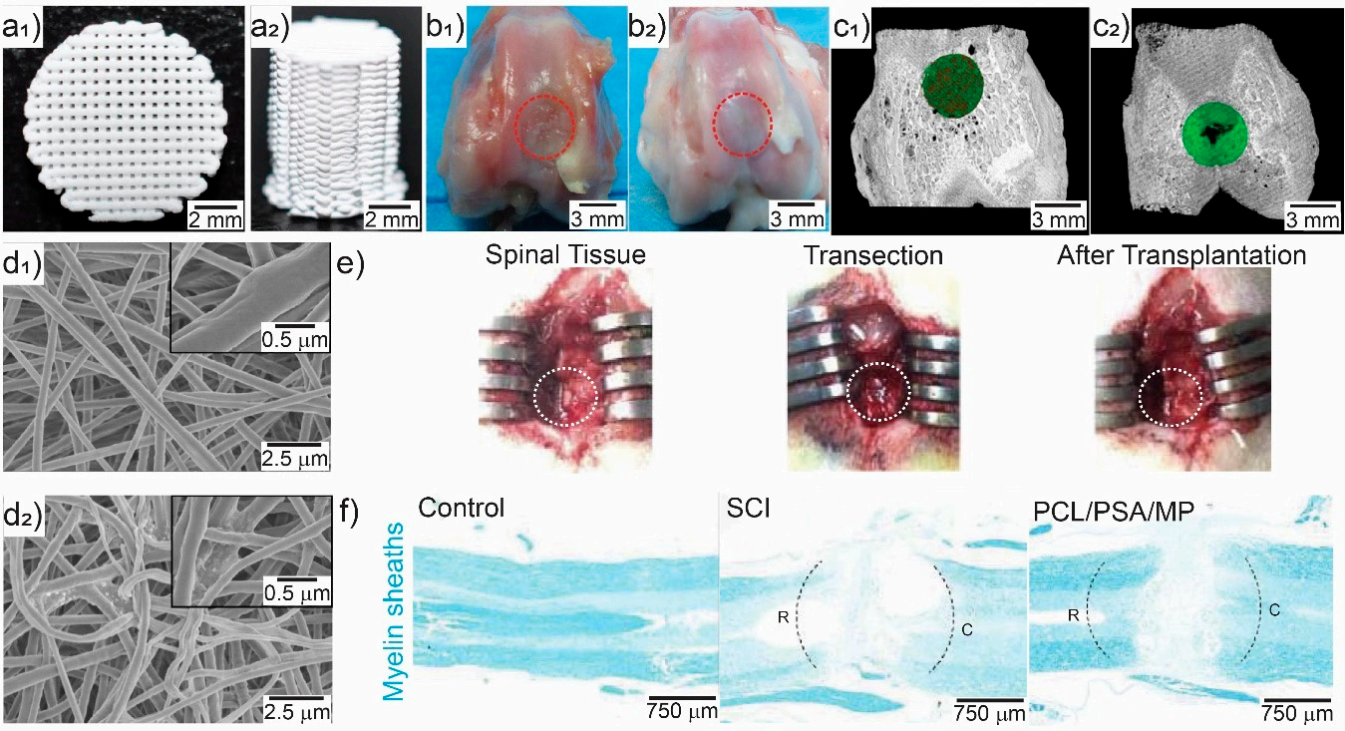
Scaffolds combining synergistic biochemical cues for TERM. (**a_1_**,**a_2_**) Gross morphology and microstructure of Li- and Si-containing scaffolds. (**b_1_**–**c_2_**) In vivo osteochondral regeneration efficiency for Li- and Si-containing scaffolds 12 weeks postsurgery: macrophotograph (**b_1_**,**b_2_**) and transverse view of 3D reconstruction of microcomputed tomography (**c_1_**,**c_2_**) showing the defects in control (untreated) (**b_1_**,**c_1_**) and implanted with scaffold (**b_2_**,**c_2_**). In microcomputed tomography the off-white color, green color and red color stand for primary bone, new bone, and scaffold, respectively. (**d_1_**,**d_2_**) SEM morphologies of untreated and after degradation for 4 weeks of PCL/polysialic acid/methylprednisolone nanofiber scaffolds, respectively. (**e**) Photographs of spinal tissue, spinal cord transection and spinal cord after nanofiber transplantation. (**f**) Histology and quantification of myelin sheaths 7 weeks postoperation. The dotted lines indicate the plane of injury. The letter R and C represents rostral and caudal spinal cord. SCI: spinal cord injury. (**a_1_**–**c_2_**) adapted with permission from [140], Elsevier, 2017. (**d_1_**–**f**) adapted with permission from [141], Elsevier, 2018.

The delivery of bioactive ions is a common strategy in the development of biomaterials for tissue engineering. In reference [140], Deng et al. presented a lithium (Li)- and Si-containing alginate scaffold fabricated via 3D-printing method (Figure 2a). The dual release of Li and Si ions from the scaffold stimulated the proliferation and maturation of chondrocytes and the differentiation of rabbit MSCs into a osteogenic lineage. The ion containing alginate scaffold exerted a positive effect on both cartilage and subchondral bone regeneration in a rabbit osteochondral defect model (Figure 2b,c). In another dual ion delivery approach, Gritsch et al. combined Cu^2+^-chitosan derivative and Sr^2+^-substituted HA into freeze-dried composite scaffolds for bone tissue engineering purposes [87]. The biomaterial showed a burst release of antibacterial Cu^2+^ with a sustained release of osteoinductive Sr^2+^. These scaffolds with adequate HA concentration presented no toxicity in MG-63 human osteoblast-like cell line. Cu^2+^ ions release has been also the focus of study in reference [86], where Jaidev et al. developed a multifunctional nanocomposite for bone tissue engineering using reduced graphene oxide (GO) coated with Cu nanoparticles in a PCL matrix. A steady release of Cu^2+^ ions was found in comparison to the burst release from the composite containing only Cu nanoparticles. The multifunctional nanocomposites were nontoxic to SVEC4-10 mouse endothelial cells and MC3T3-E1 mouse preosteoblasts, enhanced angiogenic activity (evidenced by augmented tube formation and expression of angiogenic markers), increased mineralization and bactericidal effect (against *E. coli*) in comparison with neat PCL scaffolds.

Incorporating various therapeutic molecules such as antibiotics and growth factors within the scaffold has been also explored as means to enhance tissue engineering functionalities. For example, Escobar et al. reported the use of mesoporous titania films for gentamicin loading and release to prevent Staphylococcus aureus colonization. In addition, these mesoporous materials were surface functionalized with BMP-2 to promote MC3T3 preosteoblastic cell line proliferation and differentiation [142]. In reference [141], Zhang et al. developed a multifunctional nanofiber scaffold encapsulating glucocorticoid methylprednisolone as an immunomodulatory drug for treatment of spinal cord injury. Using the electrospinning technique, the authors combined PCL and polysialic acid (a natural, biodegradable polysaccharide capable of controlling central nervous system development by modulating cell adhesion and promoting axonal growth), and methylprednisolone (a drug capable of inhibiting spinal cord early secondary inflammation and lipid peroxidation). The nanofiber scaffold was biodegradable (Figure 2(d_1_,d_2_)), biocompatible and could sustain the local release of methylprednisolone, which would bypass the severe adverse effects of high systemic dosage. Scaffolds were transplanted into spinal cord transection lesion sites in rat models (Figure 2e). The nanofiber scaffolds were effective in suppressing tissue acute inflammation (decreased levels of tumor necrosis-α and interleuquin-6), apoptosis, and attenuating glia scar formation. Moreover, the scaffolds inhibited axonal demyelination, promoted axonal regeneration, and improved functional outcome (Figure 2f).

The incorporation of different GFs within scaffolds has gain popularity over the last years, as these bioactive molecules control specific cell activities and behaviors. Wang et al. utilized the layer by layer assembly technique to develop a biomimetic extracellular matrix composed of positively charged chitosan, negatively charged oxidized sodium alginate grafted with different cell-adhesive peptides, and positively charged bovine serum albumin based nanoparticles loaded with BMP-2 [143]. While RGD adhesive peptides and penta-peptide glycine-arginine-glycine-aspartate-serine (GRGDS) grafted polyelectrolyte multilayers improved bone marrow stem cells (BMSCs) adhesion and proliferation, the addition of nanoparticles granted a porous nanostructure to the multilayer facilitating the sustained release of BMP-2, which promoted BMSCs function and differentiation into an osteogenic lineage. In vivo studies in rabbits showed the synergistic effect of GRGDS and BMP-2 scaffolds in promoting new bone tissue formation. A different approach was reported by Nih et al., where an injectable and degradable RGD-functionalized hyaluronic acid hydrogel containing heparin nanoparticles and nanoparticle-clustered VEGF was fabricated [144]. In this synergistic approach, the hyaluronic acid hydrogel promoted neuronal differentiation and the codelivery of heparin nanoparticles with anti-inflammatory properties, while the nanoparticle-clustered VEGF with angiogenic properties helped neuronal tissue repair and function recovery in a mouse brain stroke model.

### 4.3. Synergistic Approaches Combining Physicochemical and Biochemical Cues

The combination of appropriate physicochemical and biochemical cues in a biomaterial or scaffold has also led to enhanced therapeutic outcomes in tissue engineering. Ren et al. constructed composite nanofiber scaffolds composed of PLGA, HA and GO to simultaneously deliver basic fibroblast growth factor (bFGF) and BMP-2 for applications in bone tissue engineering [145]. The incorporation of HA and GO to the PLGA nanofibers significantly enhanced the mechanical properties and the hydrophilicity of the scaffold. The combination of enhanced physicochemical properties with the incorporation of GFs favored MC3T3 preosteoblasts cell adhesion. Moreover, hydrophilicity improved the immobilization of BMP-2 and bFGF which synergistically boosted cell proliferation and osteogenic differentiation (increased alkaline phosphatase activity, mineralization, and osteogenesis related gene expression). Being able to obtain the desired cell phenotype is of utmost importance in the cell therapy field, such as the treatment of enteric neuropathies with differentiated enteric neural stem cells. In reference [146], Raghavan et al. studied the influence of ECM composition on the differentiation of primary cultures of rabbit enteric neuronal progenitor cells into different neuronal subtypes and their interaction with intestinal smooth muscle sheets. Smooth muscle cells (SMCs) were aligned uniaxially by substrate microtopography and maintained a contractile phenotype irrespective of the ECM composition. ECM solution and neurons were mixed and overlaid on the aligned SMCs monolayer, followed by gelation. Fibrillar, porous ECM hydrogels with varying composition, namely combinations of Col I, Col IV, laminin and/or heparan sulfate were tested. Matrix viscoelasticity was maintained in the range 150–300 Pa adjusting component concentration, to prevent stiffness from being a variable in neuron differentiation. In the presence of SMCs, neurons differentiated to functionally innervate the muscle. ECM composition guided differentiation towards specific neuronal subtypes, from highly cholinergic (Col I), highly nitrergic (Col IV), or balanced between the two (laminin and/or heparan sulfate). Though several subtypes present in the tissue engineered intestinal sheets were capable of mediating smooth muscle contraction/relaxation, the sheets containing laminin and/or heparan sulfate showed the best balance of contractile and relaxant motor neurons.

In a different approach, to achieve antibacterial functions and proper mechanical properties for bone tissue engineering, Shuai et al. proposed a codispersion of intercalated GO nanosheets and silver (Ag) nanoparticles introduced into PLA/poly glycolic acid (PGA) 3D scaffolds prepared by additive manufacturing technology [147]. The codispersion of nanomaterials showed a synergistic effect on antibacterial efficiency by combining the capturing effects of GO nanosheets and the toxicity for bacteria coming from the effects of Ag^+^ ions released from the nanoparticles. A complementary effect in enhancing mechanical properties was also observed. The scaffolds presented good adhesion and proliferation of MG-64 cells (human bone osteosarcoma cells). In another approach for bone repair, Martin et al. [148] developed 3D printed PLA scaffolds multifunctionalized with Col, minocycline antibiotic and bioinspired citrate-hydroxyapatite nanoparticles (nHA). The uniform macroporosity, adequate wettability and excellent compressive strength of the scaffold resemble the properties of native bone. The adequate release profile of antibiotic resulted in antibacterial activity against *S. aureus*, as seen in the agar disk diffusion test and in SEM images. The multifunctionalized scaffolds synergistically improved adhesion and proliferation, and enhanced the osteogenic commitment of human BMSCs in comparison to the scaffolds functionalized with only Col. Though chitosan-based hydrogels are promising tools in TERM due to their superior biocompatibility, their weak mechanical properties hinder their broad application. In reference [149] the authors fabricated multifunctional chitosan hydrogels by reinforcing their mechanical properties with different amounts of cellulose nanocrystals (CNCs) and loaded the scaffold with tetracycline to achieve antibacterial properties. Both composite and pure chitosan scaffolds were hemocompatible. The scaffold with CNCs presented an increase in mechanical strength (modulus), a decrease in toughness and improved viability, osteogenic-related gene expression, and mineralization of BMSCs compared to pure chitosan scaffolds. Moreover, tetracycline release was more sustained in the composite scaffolds achieving an enhanced antibacterial activity against Bacillus subtilis.

In addition to enhanced mechanical properties, tailored topographical cues have been also combined with biochemicals to achieve synergistic effects in tissue engineering. In reference [150], Luo et al. designed core/shell structures of alginate/HA composite with enhanced protein release and optimal mechanical, pore and surface properties for bone tissue engineering. Alginate porous scaffolds were prepared using 3D plotting, and then, a HA nanoshell layer was achieved by in situ mineralization under mild conditions (room or physiological temperature and without any organic solvent). Pure alginate and mixed alginate/HA scaffolds (prepared by mixing alginate with HA powder) were used as controls. The surface mineralization enhanced the mechanical properties of the scaffold in comparison to pure alginate ones. The core/shell scaffolds also presented more human BMSCs adhesion, spreading and alkaline phosphatase activity than the controls. Furthermore, the scaffolds with surface mineralization presented the best sustained protein release and the mild conditions of mineralization would allow drugs and GFs loading during biomaterial preparation without denaturation. In [151] Boroujeni et al. combined topographical and biochemical cues to stimulate the differentiation of human Wharton’s jelly-derived MSCs into SMCs. Using the electrospinning technique, they fabricated aligned scaffolds made of PCL containing TGF-β1-loaded chitosan nanoparticles and PLA. The nanofiber topography and the sustained release of TGF-β1 resulted in a synergistic effect on stem cell differentiation with increased expression of SMCs markers. In reference [152], Xu et al. combined aligned electrospun nanofibers and bioglass (BG) ionic products in cell culture medium to activate a co-cultured skin cell model of human dermal fibroblasts (HDFs) and human umbilical vein endothelial cells (HUVECs). The authors found that compared to single biomaterial structural or chemical signals, the combination of these signals synergistically promote differentiation of HDFs trough stimulation of gap junctional communication and promotion of paracrine effects. While both aligned electrospun nanofibers and BG ionic products (principally Si^4+^) enhanced paracrine effects, gap junctional communications between HDFs and HUVECs were only stimulated by the structural signals. Through stimulation of vascularization and ECM protein synthesis, the activated skin tissue engineered constructs significantly improved wound healing in vivo in comparison to control experiments (wounds left untreated and wounds treated with human epidermal growth factor gel were negative and positive controls, respectively). Moreover, in reference [153] the authors synthesized gelatin/beta-tricalcium phosphate (β-TCP) 3D nanocomposite scaffolds via the solvent casting method for bone defect regeneration purposes. The scaffold combined the porous structure of gelatin (50–200 µm) with the mechanical reinforcement and osteoconductivity properties of the 90 nm diameter β-TCP spherical nanoparticles. Osteosarcoma cells (G-292) presented an enhanced rate of proliferation with higher β-TCP nanoparticles concentration. Nanocomposite samples were loaded with zoledronic acid (a drug used to treat osteoporosis), which further increase G-292 proliferation. The composite scaffolds implants in rabbit’s calvarial defects showed new bone formation and blood vessels generation, which has the potential to be a better choice in bone graft replacements than commercially available technologies.

**Figure 3:**
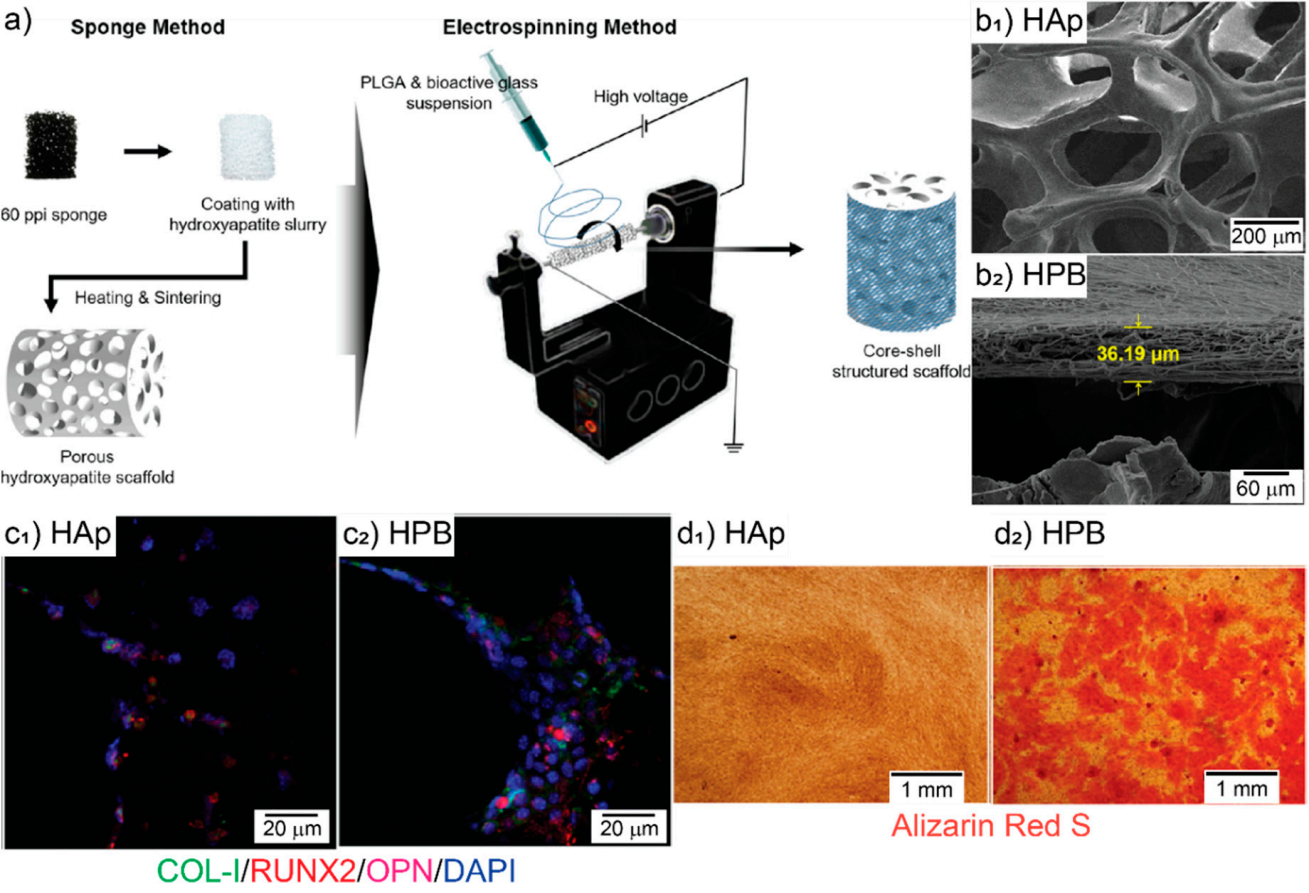
Scaffold combining synergistic physicochemical and biochemical cues for TERM. (**a**) Schematic representation of the fabrication of porous HA scaffolds covered with a BG-containing PLGA fiber layer (HPB). (**b_1_**,**b_2_**) Representative SEM images of porous HA and HPB scaffolds, respectively. The thickness of BG-containing PLGA microfibers is shown. (**c_1_**,**c_2_**) Osteogenic activity of MC3T3-E1 cells cultured on HA and HPB scaffolds, respectively. Immunocytochemical analyses of the osteogenic protein expression level of Col I (COL-I, green), runt-related transcription factor 2 (RUNX2, red) and osteopontin (OPN, magenta) following culture for 14 days. (**d_1_**,**d_2_**) Alizarin red staining performed to observe mineralization of MC3T3-E1 cells by ions released around HA and HPB scaffolds, respectively, on day 21. (**a**–**d_2_**) adapted with permission from [154], American Chemical Society, 2019. Further permissions to the related material should be directed to the ACS.

In another approach combining topographic cues and ions release, Ryu et al. developed porous HA scaffolds coated with PLGA/45S5 BG composite microfibers for bone tissue engineering [154]. The scaffold was fabricated using the sponge replica method and the thickness of the BG-containing PLGA microfibers coating was controlled via the electrospinning process time (10, 20 and 30 min) (Figure 3a). The microfiber coating enabled controlled release of Si^4+^, Ca^2+^, Na^+^ and P^3−^ ions from the scaffold for up to 28 days. Good viability and proliferation were observed for all the scaffolds and higher levels of osteogenesis-related markers and mineralization was found on 20 and 30 min electrospun coated scaffolds (Figure 3(b_1_–c_2_)). Highly anisotropic 3D structures that mimic native tissue complexity are described by Canadas et al. [155]. The authors described polymeric 3D structures of methacrylated gelatin and gellan gum that combine a linear/random porosity. The porosity was controlled through ice templating with a gradient distribution of HA formed by convection streams induced by temperature differences in the mixing of the two polymeric solutions. Moreover, a gradient of GFs was generated in the culture media using a bioreactor device that perfused either basal or osteogenic (proangiogenic) medium. Fat pad adipose-derived stem cells were seeded in the scaffold and spatially controlled osteogenic and chondrogenic markers expression was induced, generating a 3D osteochondral tissue model. Additionally, by seeding also human adipose microvascular endothelial cells, prevascularization was spatially induced in the bone-like regions. This platform has potential applications in the study of heterotypic tissues, drug delivery and tissue engineering.

Natural, tissue-specific ECM can be obtained by decellularization of organs and tissues via chemical, enzymatic or mechanical disruption [156,157]. Re-seeding and repopulation of decellularized ECM is a promising approach to generate bioartificial organs. The development of perfusion decellularization allowed the obtention of scaffolds with higher structural organization, making possible to retain the structural functions of the ECM as well as the biological ones [156]. Novel protocols and techniques are needed to overcome the difficulties in the decellularization process of specific tissues and organs, such as the high lipid content of human pancreas. Sackett et al. developed a decellularization and delipidization protocol of human pancreatic tissue for the production of ECM hydrogel scaffold that sustains cell growth and viability both in vitro and in vivo environments [157]. Though decellularized ECM scaffolds often exhibit superior biocompatibility and induce favorable immune response, the lack of hierarchical porous structures that guide directional migration and spatial organization limit their use in oriented tissues such as muscle, nerve and artery. To address this problem, Zhu et al. engineered ECM scaffolds with parallel microchannels by subcutaneous implantation of sacrificial PCL microfibers templates, followed by removing of the polymeric template and decellularization [158]. ECM scaffolds presented cell guiding effects in vitro and enhanced cell infiltration and vascularization upon in vivo implantation. Moreover, by designing and fabricating different sacrificial templates, regeneration of innervated and vascularized neomuscle (membranous ECM with longitudinally aligned microchannels), vascularized neonerve (tubular scaffolds with longitudinally oriented microchannels within the walls and luminal surfaces) and pulsatile neoartery (tubular ECM with circumferentially oriented microchannels in the wall) was demonstrated.

A brief summary of multifunctional scaffolds for tissue engineering reviewed in this section is presented in Table A2.

## 5. Multifunctional Scaffolds Combined with Other Therapies

Endowing multifunctional properties to a scaffold by combining physicochemical and biochemical cues has resulted in improved tissue engineering approaches. However, those synergistic approaches alone often lack success in tissues regeneration and integration in vivo, which ultimately limits their clinical translation. To overcome this limitations, more sophisticated approaches have been explored by combining multifunctional scaffolds with other aiding therapies as it will be highlighted in this section.

### 5.1. Synergistic Approaches Combining Multifunctional Scaffolds with Cell-Based Therapy

**Figure 4:**
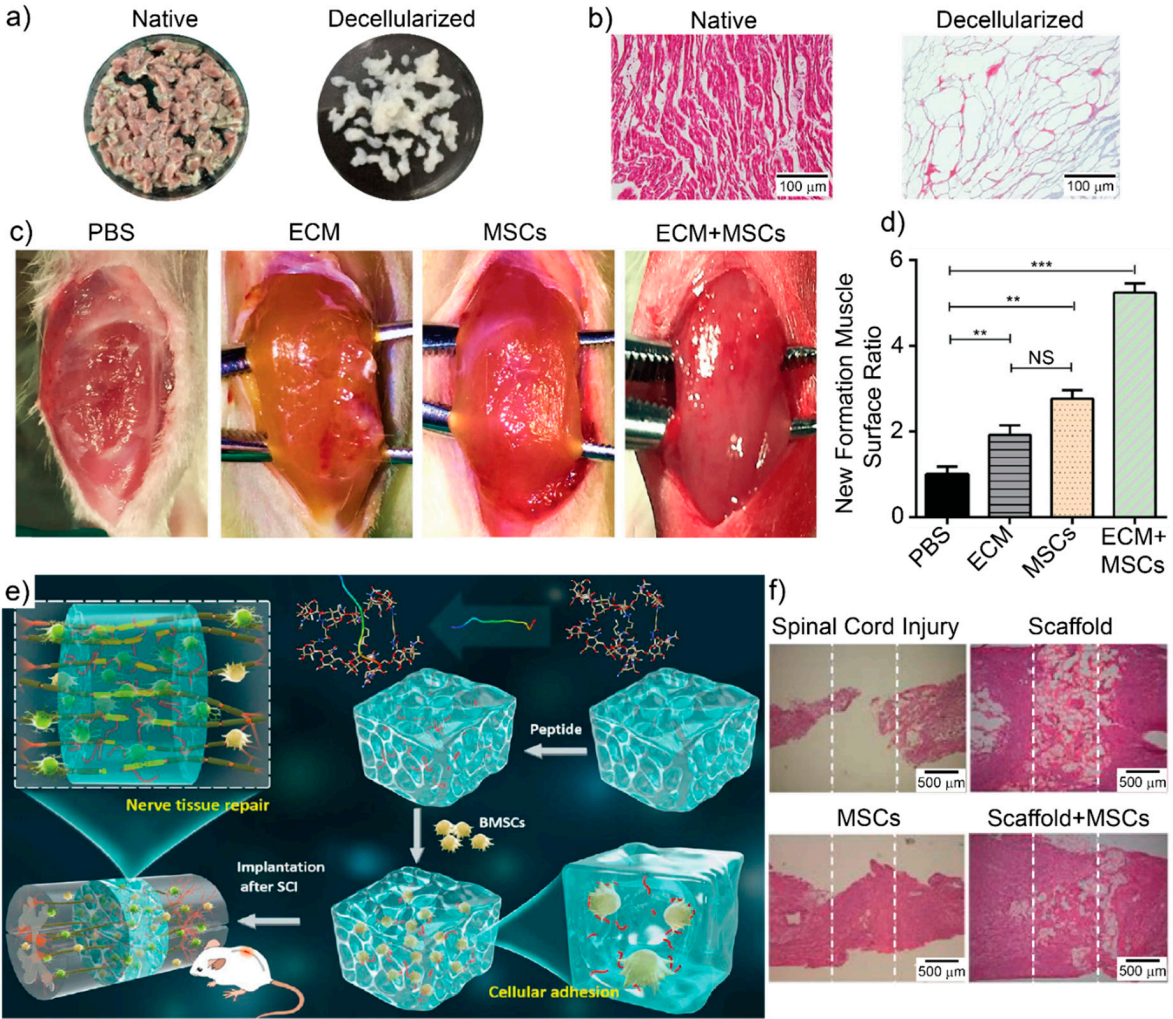
Synergistic approaches combining multifunctional scaffolds with cell-based therapy for TERM. (**a**) Representative optical images of native and decellularized heart tissue. (**b**) Masson’s trichrome staining confirming absence of cells and cell debris in the matrix after decellularization. (**c**) Gross appearance of the volumetric muscle loss injury in four groups and (**d**) surface ratio of new muscle fibers formed in each group. MSCs and ECM scaffold have a synergistic effect promoting muscle tissue regeneration. Data shown as mean ± SD. ** *p* < 0.01; *** *p* < 0.001; NS, not significant (*p* > 0.05). PBS phosphate buffer saline. (**e**) Scheme of the adhesive peptide modified hyaluronic acid scaffold loaded with bone marrow MSCs for spinal cord injury repair. (**f**) H&E staining of nontreated tissues (spinal cord injury) and tissues embedded with implants at 4 weeks postsurgery. Encapsulation of MSCs significantly improved nerve tissue reconnection effect of the scaffold. (**a**–**d**) adapted from [159], BioMed Central, 2018. (**e**–**f**) adapted with permission from [161], American Chemical Society, 2017.

The design of multifunctional decellularized scaffolds (natural tissues from which cells have been removed) combined with cell-based therapy has been reported as successful approach to aid tissue regeneration and integration. In reference [159], decellularized ECM scaffolds from cardiac tissue (Figure 4a,b) were used in combination with human umbilical cord MSCs to promote skeletal muscle regeneration in a volumetric muscle loss model. The authors proposed that the decellularized ECM scaffold and MSCs collaboratively regulate macrophage polarization toward M2 phenotype changing the default response to injury and facilitating a constructive remodeling outcome (Figure 4c,d). In another approach combining decellularized ECM, stem cells, and GFs, Farnebo et al. developed a biodegradable hydrogel for tendon-tissue engineering [160]. Tendons from cadaveric forearms were decellularized and the obtained ECM solution was supplemented with varying concentrations of bFGF, insulin-like growth factor–1 (IGF-1), and platelet-derived growth factor–BB (PDGF-BB). The gel solution was mixed with adipose-derived stem cells (ASCs) and seeded on multi-well plates or injected into the back of Sprague Dawley rats. The authors found that the GFs synergistically improved ASCs proliferation. Moreover, in vivo experiments showed that ASCs stimulates endogenous repopulation of the gel.

Cell therapy can also be combined with multifunctional synthetic scaffolds for an enhanced therapeutic outcome. Hansen et al. used the electrospinning method to fabricate biodegradable PCL scaffolds loaded with either bFGF or connective tissue growth factor, and rat MSCs [162]. The scaffold was evaluated in a full-thickness abdominal wall defect rat model. After a histological examination, they found that the meshes delivering connective tissue GFs and rat MSCs did not present complications and improved the biochemical and biomechanical properties of the weakened abdominal wall. Though implantation of biomaterial scaffolds and stem cells is often studied in animal models, very few investigations have reported the comparative study among them. An example of comparative studies is reported by Li et al., where they developed a hyaluronic acid scaffold modified with an adhesive peptide capable of promoting MSCs adhesion and survival (Figure 4e) [161]. The implantation of both, scaffold and MSCs, synergistically promoted spinal cord transection recovery (Figure 4f), helping restoring locomotor function and reducing the inflammatory response.

### 5.2. Synergistic Approaches Combining Multifunctional Scaffolds with Gene Therapy

**Figure 5:**
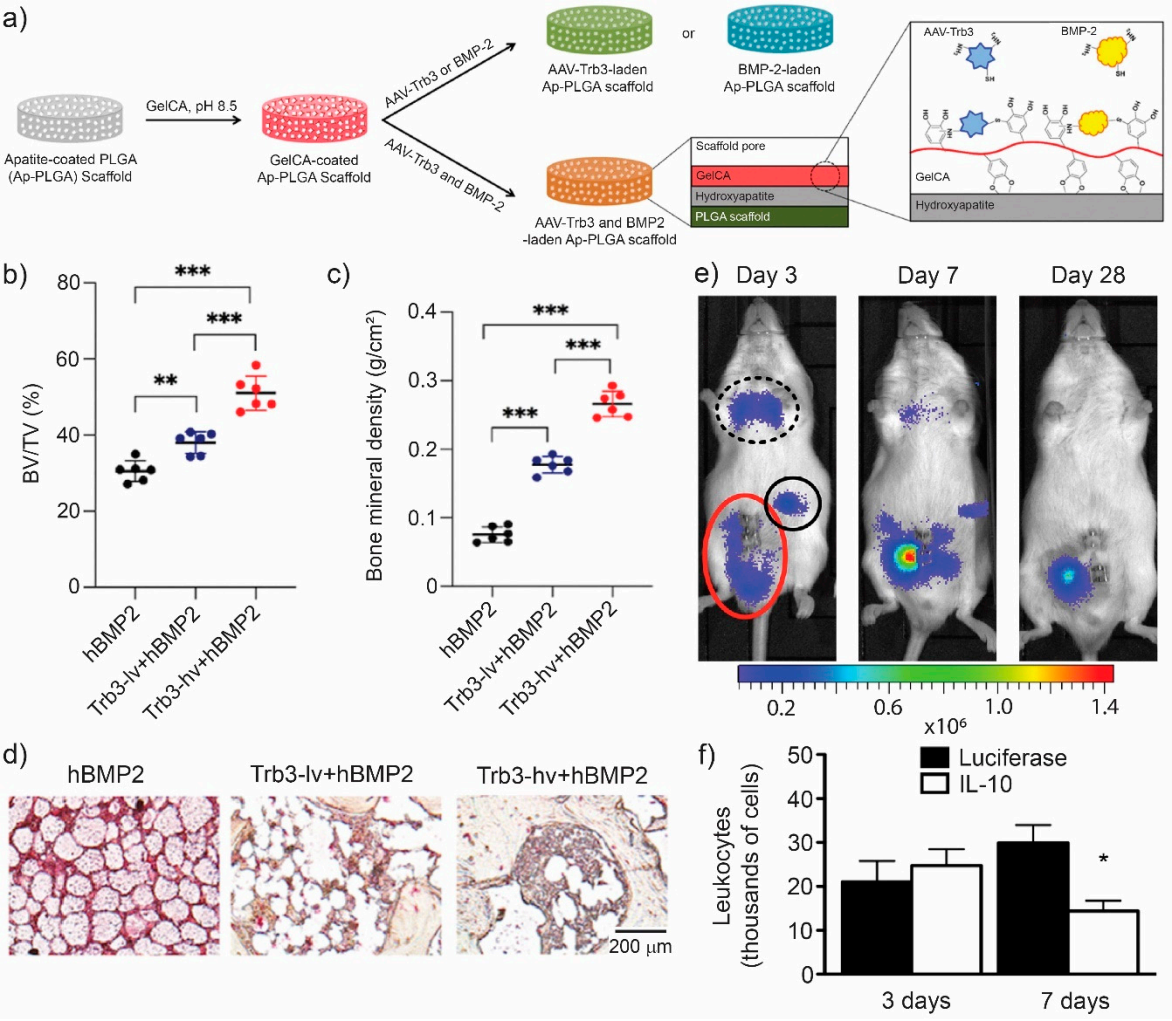
Synergistic approaches combining multifunctional scaffolds with gene or immune therapy for TERM. (**a**) Schematic diagram of scaffold fabrication through coating gelatin-conjugated caffeic acid (GelCA) onto the surface of apatite-PLGA (Ap-PLGA) scaffold. The GelCA-coated hybrid biopolymer scaffold was adopted to deliver adeno-associated viruses-encoding Trb3 or/and recombinant BMP-2 protein. (**b**–**d**) Promotion of bone formation (**b**,**c**) and fat-filled cyst formation inhibition in rat critical-sized mandibular defects 12 weeks after scaffold implantation loaded with high dose BMP-2 (hBMP2) and/or low and high dose adeno-associated viruses-encoding Trb3 (Trb3-lv and Trb3-hv, respectively). (**b**) Bone volume/tissue volume percentage. (**c**) Bone mineral density. Values represent mean ± SD: ** *p* < 0.01 and *** *p* < 0.001 using one-way ANOVA test. (**d**) Oil red immunohistochemical stain showing fewer adipocyte-like cells in area of defects treated with high dose AAV-Trb3 and BMP-2 than with BMP-2 alone. (**e**) Transgene expression in vivo. Representative bioluminescence images at days 3, 7, and 28 postimplantation of scaffolds loaded with luciferase lentivirus and implanted into the right intraperitoneal fat pad. Color bar indicates radiance (p/sec/cm^2^/sr). (**f**) Leukocyte infiltration into IL-10 virus releasing scaffolds. Number of CD45 positive cells isolated from scaffolds loaded with IL-10 or luciferase virus at day 3 and day 7 post implantation. * *p* < 0.05 versus day 7 luciferase. (**a**–**d**) adapted with permission from [164], Elsevier, 2020. (**e**–**f**) adapted with permission from [165], Elsevier, 2013.

Gene therapy has also been explored in combination with multifunctional scaffolds. Due to their similarities to the bone ECM, Col/calcium phosphate scaffolds are appealing for bone reconstruction. Additionally, calcium phosphate can be used as a nonviral vector for gene delivery as it forms complexes with plasmid DNA. Keeney et al. employed Col/calcium phosphate scaffolds to deliver a naked therapeutic plasmid encoding VEGF (pVEGF) to promote angiogenesis, and consequently bone formation, in a mouse model with intra-femoral defects [163]. In comparison to the gene-free scaffold, a twofold increase in bone volume at the defect site was measured with the delivery of pVEGF. In reference [164], Fan et al. employed scaffold-mediated local Trb3 gene delivery, a key molecular switch that controls adipocyte-osteoblast differentiation in MSCs, for bone tissue engineering. PLGA scaffolds were fabricated via solvent casting and leaching methods and coated with an apatite layer. The scaffold was subsequently functionalized with gelatin-conjugated caffeic acid loaded with BMP-2 and/or adeno-associated viruses encoding Trb3 (Figure 5a). This combined strategy stimulated robust bone regeneration (Figure 5b,c) and inhibited fat-filled cyst formation (Figure 5d) in a rodent nonhealing mandibular defect model.

Angiogenesis is key in promoting functional healing and graft integration with the host tissue. Soft tissues such as skin are richly innervated and Schwann cells (SCs) play angiogenic roles that could assist wound healing. Laiva et al. fabricated gene-activated scaffolds to drive SCs differentiation and promote angiogenesis [166]. Freeze-dried porous Col-chondroitin sulfate scaffolds were fabricated and crosslinked to provide structural reinforcement. Polyplex nanoparticles were formulated by mixing the cationic polymer vector polyethyleneimine (PEI) with plasmid DNA encoding stromal-derived factor-1α (SDF-1α), a proangiogenic chemokine gene. The polyplexes were soak loaded onto the freeze-dried scaffolds. SCs seeded on the scaffolds presented differentiation towards a repair phenotype, enhanced the production of bioactive VEGF that can significantly promote endothelial angiogenesis and ECM remodeling with laminin enrichment in comparison to the gene-free scaffold. Moreover, in reference [167], Curtin et al. developed a bioactive, Col/nHA scaffold as a platform for combinatorial gene therapy in bone regeneration. nHA was used to increase the mechanical properties of the scaffolds and also as nonviral vector for plasmid DNA delivery of pro-osteogenic BMP and proangiogenic VEGF genes. These dual combinatorial gene-activated scaffolds increased vascularization and bone repair by host cells in a rat’s cranium trans osseous defect where the implants were inserted. Gene therapies can be performed ex vivo, by transfecting cells in vitro (outside of the host), and then implanting those cells in the targeted organ (tissue). In reference [168], Qu et al. utilized an scaffold-based ex vivo gene therapy approach for bone regeneration in a rat calvarial critical-sized defect model. Rat BMSCs were transfected with bFGF (an inducer of angiogenesis and bone repair) using lipofectamine. Then, the transfected cells were seeded on porous nHA/polyamide 66 scaffolds, which are known to have excellent biocompatibility and mechanical properties. The bFGF-mediated ex vivo gene transfer based on BMSCs accelerated vascularization and bone regeneration, as shown by increased micro vessel density and new bone volume in comparison to nontransfected BMSCs-scaffold composite.

Gene therapy has been also combined with cell therapy and multifunctional scaffolds to enhance tissue regeneration. In reference [169], the authors developed gene-activated alginate hydrogels to control the differentiation of BMSCs for either cartilage or endochondral bone tissue engineering. nHA was employed as a non-viral gene transfer. Alginate hydrogels were loaded with BMSCs and nHA complexed with plasmids encoding for BMP-2 and/or transforming growth factor β3 (TGF-β3). While an increased glycosaminoglycan and Col production was found in the codelivery group in comparison to the solo delivery of plasmid BMP-2 or plasmid TGF-β3, greater levels of calcium deposition were observed with the solo gene delivery. BMSCs differentiation can be directed toward a chondrogenic or osteogenic phenotype depending on whether plasmid BMP-2 or plasmid TGF-β3 were delivered in combination or individually.

### 5.3. Synergistic Approaches Combining Multifunctional Scaffolds with Immune Therapy

Apart from cells, genes and cytokines, the intrinsic physicochemical properties of the scaffolds can modulate the immune response [121]. For instance, Knopf-Marques et al. aimed to design poly-L-lysine and hyaluronic acid polyelectrolyte multilayer films to decrease immune reactions that could lead to implant rejection [170]. They found that chemical modification of hyaluronic acid with aldehyde moieties allow self-cross-linking of the film, improving mechanical properties and guiding monocyte polarization towards an anti-inflammatory, prohealing phenotype. This effect was further increased with film loading and release of immunomodulatory cytokine (IL-4). Garg et al. employed polydioxanone electrospun scaffolds and found that pore size is a regulator of bone marrow-derived macrophages polarization towards regenerative (M2) or inflammatory (M1) phenotypes [171]. By controlling scaffold properties, they induced a M2-like profile capable of promoting angiogenesis in a 3D in vitro bead assay. In an attempt to prevent foreign-body reaction and capsule formation that would impede the performance of implantable biomedical devices, Zhang et al. developed ultra-low-fouling zwitterionic hydrogels that can resist capsule formation after subcutaneous implantation in mouse [172]. Moreover, the zwitterionic hydrogels also promote prohealing polarized macrophages and angiogenesis in surrounding tissue.

Multifunctional scaffolds and immune therapy have been also explored in the presence of cells. Tendon injury is a common problem with slow healing and scar formation that results in compromised function. In reference [173], Aktas et al. developed an immunomodulatory therapy combining a PLGA scaffold fabricated by a salt fusion/solvent casting/salt leaching technique, with tumor necrosis factor-alpha (TNF-α) primed MSCs to repair rat Achilles segmental defects. The TNF-α-primed MSCs-seeded PLGA scaffolds modulated macrophage polarization towards a M2 phenotype and cytokine production towards an anti-inflammatory environment, increased the concentration of type I procollagen in the healing tissue and improved strength of the tendon in comparison to PLGA scaffold without MSCs.

Multifunctional scaffolds in combination with immune therapy may be applied to treat diseases such as cancer. Antibody-based therapy has promising applications for tumor cell killing but is limited by the burst release of antibodies and their diffusion, and by slow healthy tissue regeneration after tumor clearance. In reference [174], Liu et al. developed specific immunological tissue engineering scaffolds by dopamine coating and further agonistic mouse anti-human CD40 antibody coating onto PLA electrospun fibers. The scaffolds were able to kill CD40-expressed tumor cells directly by inducing apoptosis and indirectly by dendritic cell activation. Moreover, they were able to support adhesion and proliferation of mouse preosteoblast MC3T3-E1 cells.

As biomaterial implantation may result in an inflammatory response that can impair integration with the host and tissue regeneration, Gower et al. developed a scaffold for lentiviral gene therapy and the localized delivery of anti-inflammatory IL-10 [165]. PLGA microsphere scaffolds were loaded with lentiviral vector carrying DNA encoding for IL-10. The scaffolds were implanted in the intraperitoneal fat. Expression persisted at the scaffold up to 4 weeks and macrophages were the most common leukocyte transfected (Figure 5e). Delivery of the IL-10 encoding vector significantly decreased leukocyte infiltration (Figure 5f) and decreased pro-inflammatory IFN-γ expression, thus reducing inflammation.

### 5.4. Synergistic Approaches Combining Multifunctional Scaffolds with Energy-Based Therapy

Another success synergistic approach has been found by combining multifunctional scaffolds with electrical, magnetic, or light-based therapies. Jin et al. fabricated 2D aligned conductive nanofibers by electrospinning PLA and subsequently polymerizing on the surface conductive PEDOT [175]. The authors reported a synergistic effect of the aligned nanopattern and the electrical stimulation on human MSCs growth behavior, maintaining cellular activity, promoting contact between cells and improving tissue-like formation. Rao et al. developed magnetically responsive polyelectrolyte hydrogels via self-organization of xanthan gum and chitosan in the presence of iron oxide MNPs [176]. The incorporation of the MNPs greatly improved the mechanical properties of the hydrogels. Moreover, NIH3T3 fibroblasts seeded on magnetic hydrogels presented enhanced adhesion properties and proliferation in presence of an external magnetic field with respect to pristine polyelectrolyte complex hydrogels.

**Figure 6:**
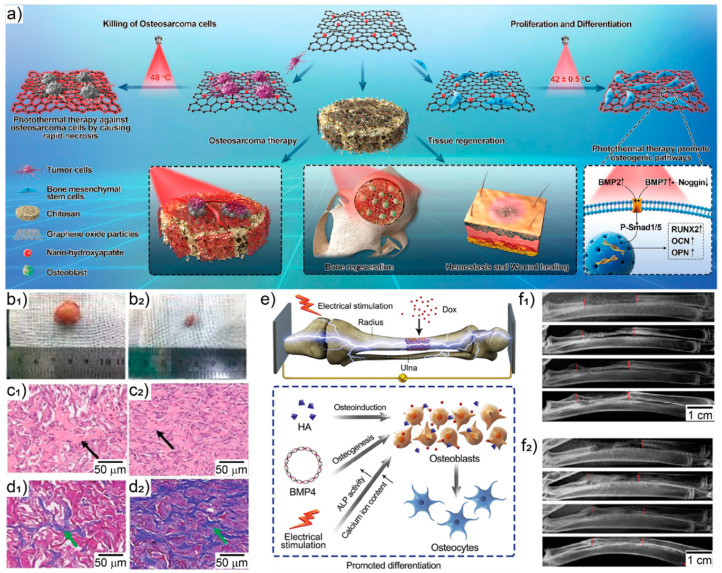
Synergistic approaches combining multifunctional scaffolds with energy-based therapy for TERM. (**a**) Schematic illustration of the fabrication of nHA/GO particles, nHA/GO/chitosan scaffolds, and their bioapplication. (**b_1_**,**b_2_**) Representative tumor photographs of control group (without adding any scaffolds or irradiation) and nHA/GO/chitosan scaffold implanted and daily irradiated tumors, respectively. (**c_1_**–**d_2_**) H&E and Masson histological analysis of the new bone formation (black arrows, new bone; green arrows, collagen fiber). (**c_1_**,**d_1_**) control group. (**c_2_**,**d_2_**) irradiated nHA/GO/chitosan scaffolds. (**e**) Construction of a smart electroactive tissue engineering scaffold with ability to control release and expression of BMP-4 for efficient bone repair. (**f_1_**,**f_2_**) In vivo repair of smart electroactive polymer scaffolds. X-ray detection of rabbit radial defect implanted with PLGA/HA scaffold without electrical stimulation (**f_1_**) and PLGA/HA/PLA-aniline pentamer/BMP-4 scaffold with electrical stimulation (**f_2_**). Four images from top to bottom were collected in 2-, 4-, 8-, or 12-weeks postoperation for each group. (**a**–**d_2_**) adapted with permission from [177], Elsevier, 2019. (**e**–**f_2_**) adapted with permission from [179], Elsevier, 2019.

The therapeutic outcome of certain diseases in which TERM therapies are crucial, often is negatively impacted by underlying diseases. For instance, large bone defects caused by a tumor osteosarcoma resection can be treated with materials that induce bone regeneration, but generally these materials do not destroy residual tumor cells neither promote soft tissue repair. To address these issues, Ma et al. developed a multifunctional nHA/GO/chitosan scaffold with photothermal activity [177]. In vitro experiments showed that under 808-nm near-infrared light irradiation, the scaffold can kill human osteosarcoma cells (when reaching temperatures of 48 °C), or promote osteogenesis of human BMSCs (when reaching temperatures of 42 °C) in coordination with the osteogenic properties of nHA (Figure 6a). The irradiated scaffolds presented good antitumor activity in osteosarcoma tumor-bearing mice (Figure 6(b_1_,b_2_)). Moreover, the combined therapy (scaffold + irradiation) showed the best postoperative bone volume/tissue volume ratio performance in cranial defects of rats (Figure 6(c_1_–d_2_)) and soft tissue wound healing ability in mice skin defects in comparison with the controls. Tissue engineering strategies can be utilized to treat diseases and simultaneously promote the regeneration of the injured tissue. Samaneh Saber-Samandari et al. designed a porous bifunctional scaffold for cancerous bone tumor treatment composed of gelatin and akermanite, with the addition of multiwall CNTs to reinforce the mechanical properties of the biomaterial and promote cell adhesion, and iron oxide MNPs to kill cancer cells via photothermal activity [178].

More sophisticated approaches have been reported by combining multifunctional scaffolds with more than one aiding therapy. In reference [180], Leppik et al. studied the combination of β-TCP scaffolds, adipose tissue derived MSCs (AT-MSCs) and electrical stimulation as a possible treatment for large bone defects. The authors found increased osteogenic gene expression and differentiation in vitro with the electrical stimulation of the AT-MSCs + β-TCP. Moreover, in vivo rat femur defects treated with AT-MSCs + β-TCP scaffold and electrical stimulation presented improved bone healing as seen by the greater amounts of new bone and vascularization, less fibrous tissue, increased osteogenic gene expression and bone strength. In another approach, Gelmi et al. developed PLGA fiber scaffolds coated with the conductive polymer polypyrrole for delivery, support and electro-mechanical stimulation of induced pluripotent human stem cells (iPCS) for cardiac tissue engineering [181]. Electrical stimulation led to reversible volume changes of polypyrrole, which generates mechanical actuation. These novel electromechanically active fiber scaffolds present no cytotoxic effects on iPCS and increased expression of cardiac markers. In an attempt to mimic the unidirectional electrical impulses of the embryonic heart during cardiogenesis, Mohammadi Amirabad et al. fabricated via electrospinning aligned PANI/polyestersulfone nanofibrous scaffolds doped by Camphor-10-sulfonic acid [104]. These scaffolds were used to support cardiovascular disease-specific iPSCs and to deliver electrical impulses in a unidirectional fashion. The application of unidirectional electrical stimulation to the cells upregulated the expression of cardiac-related transcription factors and cardiac-specific structural genes, and significantly increased the number of cardiac Troponin T cells in comparison to multidirectional electrical stimulation using random fibrous scaffolds. These scaffolds may be useful for the generation of cardiomyocytes in vitro for cell replacement therapies in cardiovascular diseases.

As GFs and other bioactive molecules usually act in a short-range diffusion trough the ECM, rather than in an endocrine fashion, scaffolds for tissue engineering are often loaded with them for delivery approaches. However, poor stability in physiological conditions, deactivation/degradation by enzymes in vivo, and difficulties in the control of dosis of these bioactive molecules at the targeted tissues limit their broad clinical application in the tissue regeneration field [182]. In order to overcome these limitations, more complex strategies are being developed. In reference [179], Cui et al. described an electroactive composite smart scaffold with local osteoinductive factor expression for rapid and efficient bone repair upon electrical stimulation (Figure 6e). By addition of a human BMP-4 gene fragment to an artificial restructuring plasmid vector to form a BMP-4 plasmid, the authors achieved doxycycline-regulated gene expression. They used PEI conjugated gold nanoparticles as a plasmid carrier with high transfection efficiency. An electroactive, biocompatible and biodegradable triblock copolymer of PLA-block-aniline pentamer-block-PLA combined with a PLGA/HA matrix was employed as the stimuli-responsive bone tissue-engineering scaffold. Plasmid release from the scaffold was controlled by electrical stimulation. The composite scaffold, the gene vector complex and the electrical stimulation were demonstrated to synergistically improve cell proliferation and differentiation in vitro, and effective bone healing in vivo in a rabbit radial defect model (Figure 6(f_1_,f_2_)).

A brief summary of the combined multifunctional scaffolds with therapies for tissue engineering reviewed in this article is presented in Table A3. Moreover, a brief summary of different strategies for facilitation of cellular processes reviewed in this article is presented in Table A4.

## 6. Conclusions, Challenges and Future Perspectives

We have reviewed here a large number of possibilities explored in the fabrication of multifunctional scaffolds for TERM. The different research approaches explored comment on the complexity of TERM, and at the same time on its therapeutic relevance. Nano- and microtechnologies offer multiple possibilities for the spatiotemporal control of biomolecules delivery, the biomimicking of ECM properties, and for endowing scaffolds with multiple functionalities. A clear tendency can be inferred from the research discussed towards the combination of multifunctional materials with different therapeutic approaches: cell, gene, immune, electric, magnetic, and light-based. The combination of a multifunctional material that inherently provides a microenvironment resembling the native ECM, with the delivery of cells (cell therapy) generally results in enhanced cell proliferation, differentiation, and formation of new tissues. Combining gene delivery with multifunctional scaffolds allows to specifically manipulate cell differentiation towards the desired phenotype and to provide an adequate microenvironment to promote the vascularization and innervation of new tissues. Multifunctional scaffolds loaded with immunomodulatory agents intend to overcome challenges in tissue healing associated with scaffold/implant rejections. These combinatorial approach results in desired M2 macrophage polarization which resolves inflammation and tissue healing. Combining stimuli-responsive nanomaterials with multifunctional scaffolds allows to deliver additional mechanical stimulus or energy into the cellular microenvironment, which has been demonstrated to enhance cell proliferation, migration, and differentiation. The overall goal of combinatorial approaches is the optimization of the material–cell interactions and to create the most suitable niche for the cells to develop into a tissue or organ, i.e., appropriate angiogenesis, osteogenesis, interaction with the immune system, etc., which cannot be achieved without the synergistic effect of multiple therapies.

All the research approaches conferred in this review emphasize the need to emulate the complexity of native ECM, however, it is worth to mention a new tendency in TERM highlighting “minimalist-engineering” approaches [183]. Although combination therapy results in enhanced tissue regeneration or repair, it is known that multifunctional materials could hamper with natural cell arrangement and behavior, which is spontaneous and relies on multiple cell populations. Minimalist-engineering tries to preserve collective cell self-organization with low-material strategies, in which the quantity of biomaterials used for TERM is minimized to potentially favor natural cell healing processes. However, regardless the use of complex or minimalistic approaches, the main goal is to create a suitable niche for cell growth and tissue formation.

Although TERM field has substantially grown over the last twenty years [21,184], there are still several challenges to be addressed. For instance, to truly mimic the physical properties of some native tissues, the biomaterials should be able to spontaneously heal and regenerate injuries [185]. Though intelligent self-healable hydrogels are the most promising candidates, hydrogels often have poor mechanical properties that make them unsuitable for application in stiff tissues and organs. TERM of certain tissues and organs present their own challenges. Mimicking the physical and biochemical characteristics of different cartilages zones is a major challenge in chondro-inductive materials [186]. Avoiding negative inflammatory responses and thrombogenesis while promoting endothelialization is a major issue in tissue engineered vascular grafts [187].

Despite the significant progress with in vitro and small animal studies, translation into clinical and commercial endpoints has been slow and not sufficient [188,189]. This gap can be attributed to a lack of consideration of a multitude of adoption, commercial and regulatory constraints. The biomaterials should reunite some mandatory and some optional specifications, depending on the application, such as: specific bioactivity, adapting to the constantly changing microenvironment, appropriate degradation kinetics and appropriate morphological and chemical degradation profiles, appropriate elastic/viscoelastic properties, injectable if desired, noncytotoxic, nonimmunogenic and minimally proinflammatory, etc. [188,190]. In this vein, though natural materials tend to be more biocompatible since they are able to remodel and degrade by cells, they often need mechanical reinforcement and obtaining them is often complicated, time-consuming and natural variations may challenge its reproducibility [187]. On the other hand, though synthetic materials are easier processed and reproducible, they require bioactive functionalization and careful design to achieve proper degradation, if required. Moreover, the pressure for publishing in academia can lead to components addition and complexity increase to enhance innovation. These approaches, despite being able to enhance the clinic performance, can add unnecessary complications to the regulatory FDA approval process and are unlikely to improve the sustainability of tissue engineering biomaterials [188,189]. A bedside to bench and back again approach is needed to finally fulfill TERM ultimate promise [189,191].

## Appendix A

**Table A1:** Compilation of the abbreviations utilized in this article.

Abbreviation	Definition
AT-MSCs	Adipose tissue derived MSCs
ASCs	Adipose-derived stem cells
RGD	Arginine-glycine-aspartate
bFGF	Basic fibroblast growth factor
β-TCP	Beta-tricalcium phosphate
BG	Bioglass
BMSCs	Bone marrow stem cells
BMP	Bone morphogenetic proteins
Ca	Calcium
CNTs	Carbon nanotubes
CNCs	Cellulose nanocrystals
CRISPR/Cas9	Clustered regularly interspaced short palindromic repeats -associated protein 9
Col	Collagen
Cu	Copper
ECM	Extracellular matrix
GO	Graphene oxide
GFs	Growth factors
HDFs	Human dermal fibroblasts
HUVECs	Human umbilical vein endothelial cells
HA	Hydroxyapatite
nHA	Hydroxyapatite nanoparticles
iPCs	Induced pluripotent human stem cells
IGF-1	Insulin-like growth factor–1
Fe	Iron
Li	Lithium
Mg	Magnesium
MNPs	Magnetic nanoparticles
Mn	Manganese
MSCs	Mesenchymal stem cells
MAO	Microarc oxidation
GRGDS	Penta-peptide glycine-arginine-glycine-aspartate-serine
P	Phosphorus
pVEGF	Plasmid encoding VEGF
PDGF-BB	Platelet-derived growth factor–BB
PEDOT	Poly (3,4-ethylenedioxythiophene)
PGA	Poly glycolic acid
PLA	Poly (D, L-lactic acid)
PLGA	Poly (lactic-co-glycolic acid)
PANI	Polyaniline
PCL	Polycaprolactone
PEI	Polyethyleneimine
PSS	Polystyrene sulfonate
PVA	Polyvinyl alcohol
SCs	Schwann cells
SAMs	Self-assembled monolayers
Si	Silicon
Ag	Silver
SMCs	Smooth muscle cells
Na	Sodium
SDF-1α	Stromal-derived factor-1α
Sr	Strontium
TERM	Tissue engineering and regenerative medicine
Ti	Titanium
TNTs	Titanium oxide nanotubes
TALENs	Transcription activator-like effector nucleases
TGF-β1	Transforming growth factor beta 1
TGF-β3	Transforming growth factor beta 3
TNF-α	Tumor necrosis factor-alpha
VEGF	Vascular endothelial growth factor
Zn	Zinc

**Table A2:** Summary of multifunctional scaffolds for tissue engineering reviewed in this article.

Physicochemical and Biochemical Cues	Materials	Technique	Application and Results	Ref
Mechanical properties and topography	Gelatin hydrogel, MNPs-decorated rod-shaped cellulose nanocrystals	Cross-linking chemistry	Cell alignment. Injectable hydrogel.	[133]
Surface chemistry and topography	Ti coated with Ca, P, Si and Na	MAO	Bone implant with enhanced regeneration and bone-impact contact.	[134]
Surface potential and topography	PCL	Electrospinning varying voltage polarity	Osteoblast proliferation, Col-like fiber formation and filopodia.	[136]
Surface chemistry and mechanical properties	PVA-CNT nanocomposite	Freeze drying	Osteoblast cell adhesion, proliferation, differentiation, phosphate activity, mineralization, and Col secretion.	[137]
Surface chemistry and electroconductivity	TNTs coated with PANI	Electrochemical oxidation and cyclic voltammetry	Enhance cell attachment, proliferation, and expression of osteogenic-related markers.	[135]
Surface chemistry, electroconductivity and topography	Silk coated with edged PEDOT-PSS	Electrospinning	Neuronal proliferation and differentiation.	[139]
Combined bioactive ions	Li and Si ions and Alginate	3D printing	Osteoarthritis. Chondrocyte’s proliferation and maturation, and MSCs differentiation into osteogenic lineage.	[140]
Combined bioactive ions	Cu^2+^-chitosan and Sr^2+^-HA	Freeze drying	Bone tissue engineering. Antibacterial and osteoconductive properties.	[87]
Combined bioactive ions	GO coated with Cu nanoparticles and embedded into a PCL matrix	Spin coating	Bone tissue engineering. Enhance angiogenic activity, mineralization, and bactericidal effect.	[86]
Antibiotics and GFs	Mesoporous Titania films loaded with gentamicin and BMP-2	EISA	Prevented *Staphylococcus aureus* colonization and promote preosteoblastic proliferation and differentiation.	[142]
Immunosuppressant and drug	PCL loaded with polysialic acid and methylprednisolone	Electrospinning	Spinal cord repair. Suppressed acute inflammation, apoptosis, and glia scar formation, and promoted axonal regeneration.	[141]
GFs and adhesive peptides	Chitosan, sodium alginate, bovine serum albumin nanoparticles, RGD, GRGDs and BMP-2	Layer-by-Layer	BMSCs adhesion, proliferation, and differentiation into osteogenic linage.	[143]
GFs and adhesive peptide	Hyaluronic acid, heparin nanoparticles, RGD and VEGF	Michael addition synthesis	Neuronal repair after brain stroke. Neuronal differentiation, anti-inflammatory and angiogenic properties.	[144]
Mechanical properties, surface chemistry and GFs	PLGA, HA and GO loaded with bFGF and BMP-2	Electrospinning	Bone tissue engineering. Enhanced cell adhesion, proliferation, and osteogenic differentiation.	[145]
Topography and bioactive proteins	Col I and IV, laminin, heparan sulfate and SMCs	Gelation at 37 °C	Muscle innervation and guided differentiation.	[146]
Mechanical properties and antibacterial function	GO and Ag nanoparticles loaded into PLL/PGA	Additive manufacturing	Bone tissue regeneration. Enhanced cell adhesion and proliferation.	[147]
Mechanical properties, surface chemistry and antibacterial	PLLA, Col, minocycline and nHA	3D printing	Bone repair. Antibacterial properties, enhanced proliferation, and osteogenic commitment.	[148]
Mechanical properties and antibacterial function	Chitosan, CNCs, tetracycline	Freeze drying	Enhanced antibacterial activity, mechanical properties, osteogenic-related gene expression and mineralization.	[149]
Mechanical properties and bioactive proteins	Alginate/HA	3D plotting and in situ mineralization	BMSCs improved adhesion and mineralization.	[150]
Topography and GFs	PCL, PLA and TGF-β1-loaded chitosan nanoparticles	Electrospinning	SMCs differentiation.	[151]
Topography, bioactive ions, and proteins	BG ionic products and cell culture media	Electrospinning	Skin tissue engineering. Improved wound healing.	[152]
Topography, mechanical properties, and drugs	Gelatin/β-TCP, zoledronic acid	Solvent Casting	Bone defect regeneration. Enhanced new bone formation and vascularization.	[153]
Topography and bioactive ions	HA-coated PLGA/45S5 BG	Sponge replica and electrospinning	Enhanced proliferation, differentiation towards osteogenic lineage, and mineralization.	[154]
Porosity and GFs	Methacrylate gelatin, gellan gum, HA, osteogenic GFs	Photopolymerization, ice templating and freeze drying	Prevascularized 3D osteochondral tissue constructs.	[155]
**Topography and** ECM components	PCL microfibers, in vivo engineered ECM scaffolds	Melt-spinning, decellularization	Cell guidance. Oriented tissue regeneration.	[158]

**Table A3:** Summary of combined multifunctional scaffolds and therapies for tissue engineering reviewed in this article.

Multifunctional Scaffolds Combined Strategies	Materials	Technique	Application and Results	Ref
Cell therapy	Decellularized cardiac ECM scaffolds and human umbilical cord MSCs	Chemical-based decellularization and freeze drying	Macrophage polarization towards M2 phenotype and promotion of skeletal muscle tissue regeneration.	[159]
	Decellularized tendons from cadaveric forearms, ASCs, bFGF, IGF-1, PDGF-BB	Chemical-based decellularization	Improved ASCs proliferation and endogenous repopulation.	[160]
	PCL, bFGF, connective tissue growth factor, rat MSCs	Electrospinning	Abdominal wall defect repair. Improved biochemical and biomechanical properties in abdominal wall.	[162]
	Hyaluronic, adhesive peptide, MSCs	Crosslinking	Spinal cord transection recovery, restored locomotor functions and reduced inflammation.	[161]
Gene therapy	Col/Calcium Phosphate, pVEGF	Gelation	Promote angiogenesis and bone formation in mouse intra-femoral defects.	[163]
	HA-coated PLGA, Trb3 encapsulated in gelatin-conjugated caffeic acid	Solvent casting and leaching	New bone formation, inhibited fat-filled cyst formation in a non-healing mandibular defect rodent model.	[164]
	Col-Chondroitin sulfate, PEI, SDF-1α, proangiogenic chemokine gene	Freeze drying and cross-linking	SCs differentiation and angiogenesis.	[166]
	Col/nHA, BMP, pVEGF	Freeze drying and cross-linking	Bone regeneration, increased vascularization.	[167]
	bFGF-transfected BMSCs, nHA/polyamine 66	Phase separation	Bone regeneration and vascularization in rat calvarial critical sized defect model.	[168]
	Alginate, nHA, BMSCs, plasmid BMP-2, pTGF-β3	Ionic cross-linking	Selective differentiation of BMSCs towards cartilage or endochondral bone tissue.	[169]
Immune therapy	Poly-L-lysine, hyaluronic acid, IL-4	Layer by layer and cross-linking	Decrease immune reactions in implant rejection, improved mechanical properties, guided monocyte polarization towards anti-inflammatory and pro-healing phenotype.	[170]
	Polydioxanone	Electrospinning	Induced M2-like profile that promotes angiogenesis.	[171]
	Poly (carboxybetaine methacrylate) and poly (2-hydroxyethyl methacrylate)	Photopolymerization	Prevent foreign-body reaction and capsule formation, promote healing polarized macrophages and angiogenesis.	[172]
	PLGA, TNF-α, MSCs	Salt fusion/solvent casting/salt leaching	Achilles’ tendon repair, M2 polarization, anti-inflammatory environment, increased type I procollagen.	[173]
	Decellularized MSCs	Chemical-based decellularization and freeze drying	Volumetric muscle loss, M2 polarization, skeletal muscle regeneration.	[159]
	Poly-L-lysine, dopamine, anti-CD40 antibody	Electrospinning	Cancer therapy. Kill tumor cells, support adhesion and proliferation of MC3T3-E1 cells.	[174]
	PLGA, lentivirus encoding IL-10	Gas foaming	Reduce inflammation and leukocyte infiltration.	[165]
Electrical stimulation	PLLA, PEDOT	Electrospinning	Increased MSCs growth, activity, and tissue-like formation.	[175]
Magnetic stimulation	Xanthan gum, chitosan, iron oxide MNPs	Self-organization	NIH3T3 fibroblast enhanced adhesion and proliferation. Hydrogel enhanced mechanical properties.	[176]
Photothermal stimulation	nHA/GO/Chitosan	Freeze drying and cross-linking	Treatment of osteosarcoma and tissue regeneration.	[177]
Photothermal stimulation	Gelatin, akermanite, CNTs, iron oxide MNPs	Freeze drying and cross-linking	Cancerous bone tumor treatment and bone tissue regeneration.	[178]
Cell therapy + electrical stimulation	β-TCP, AT-MSCs	Rehydration	Regeneration in large bone defects. Improved bone formation, vascularization, and less fibrous tissue.	[180]
Cell therapy + electro-mechanical stimulation	PLGA, polypyrrole, iPCs	Electrospinning	Cardiac tissue engineering, Improved expression of cardiac markers.	[181]
Cell therapy + electrical stimulation	PANI/polyestersulfone, Camphor-10-sulphonic acid, cardiovascular disease-specific iPSCs	Electrospinning	Cardiovascular diseases. Generation of cardiomyocytes.	[104]
GFs + electrical stimulation	PLA-AP, PLGA/HA, BMP-4, PEI coated gold nanoparticles	Freeze drying	Bone healing. Improved cell proliferation and differentiation.	[179]

**Table A4:** Summary of different strategies for facilitation of cellular processes reviewed in this article.

Cellular Process	Facilitation Strategies	Ref
Adhesion	Stiffness and complex stiffness	[28,137,150,178]
	Nano-micrometer surface roughness	[36]
	Fibronectin, Col, and adhesion promoting peptides	[93,94,143,161]
	Electrical stimulation	[123]
	Magnetic stimulation	[16,176]
	Surface chemistry (wettability, charge, and potential)	[51,52,53,54,56,136]
	Electroconductive surface	[135]
Alignment, recruitment, and migration	Gradients in substrate stiffness (durotaxis)	[30]
	Surface topographical cues (nanofibers, microridges, porous channels, etc.)	[38,39,40,133,158]
	Local asymmetric topographical ratchets (ratchetaxis)	[41]
	Growth factor, chemokines, and others chemical stimuli (chemotaxis)	[73]
	Electrical stimulation and electric field gradient (galvanotaxis)	[123,127]
	Magnetic stimulation	[16]
Differentiation and polarization	Stiffness guided	[31,137,149,150]
	Micropattern, nanotopography and porosity	[37,171]
	Growth factor and cytokine delivery	[72,142,143,145,170]
	Ion delivery	[83,140]
	Gene therapy (e.g., genes encoding growth factors)	[112,164,167,169]
	Electrical stimulation	[123,180]
	Magnetic stimulation	[130]
	Optical stimulation	[19]
	Electroconductive surface	[135,139]
	Bioactive polymers (e.g., hyaluronic acid) and ECM composition	[144,146,159]
	MSCs	[159]
Proliferation	Growth factor and drug delivery	[142,145,153,160]
	Ion delivery	[84,140]
	Electrical stimulation	[123,127]
	Magnetic stimulation	[16]
	Optical stimulation	[19]
	Surface chemistry (wettability, charge, and potential)	[136]
	Stiffness	[137]
	Electroconductive surface	[135,139]
	Adhesion promoting peptides	[143]
Vascularization	VEGF delivery	[71,144]
	Gene therapy (genes encoding VEGF, chemokines, etc.)	[112,114,163,166,167,168]
	Ion delivery	[86]
